# Study of smart grid cyber-security, examining architectures, communication networks, cyber-attacks, countermeasure techniques, and challenges

**DOI:** 10.1186/s42400-023-00200-w

**Published:** 2024-05-02

**Authors:** Batoul Achaal, Mehdi Adda, Maxime Berger, Hussein Ibrahim, Ali Awde

**Affiliations:** 1 Département de Mathématique, Informatique et Génie, Université du Québec à Rimouski, Allée des Ursulines, Rimouski, G5L 3A1 Canada; 2 Centre de Recherche et d’innovation en Intelligence énergétique (CR2ie), Rue De La Vérendrye, Sept-Îles, G4R 5B7 Canada

**Keywords:** Smart grid, Architecture, Communication network, Cyber attacks, Blockchain, Artificial intelligence, NIST framework

## Abstract

Smart Grid (SG) technology utilizes advanced network communication and monitoring technologies to manage and regulate electricity generation and transport. However, this increased reliance on technology and connectivity also introduces new vulnerabilities, making SG communication networks susceptible to large-scale attacks. While previous surveys have mainly provided high-level overviews of SG architecture, our analysis goes further by presenting a comprehensive architectural diagram encompassing key SG components and communication links. This holistic view enhances understanding of potential cyber threats and enables systematic cyber risk assessment for SGs. Additionally, we propose a taxonomy of various cyberattack types based on their targets and methods, offering detailed insights into vulnerabilities. Unlike other reviews focused narrowly on protection and detection, our proposed categorization covers all five functions of the National Institute of Standards and Technology cybersecurity framework. This delivers a broad perspective to help organizations implement balanced and robust security. Consequently, we have identified critical research gaps, especially regarding response and recovery mechanisms. This underscores the need for further investigation to bolster SG cybersecurity. These research needs, among others, are highlighted as open issues in our concluding section.

## Introduction

The conventional power grid is becoming a limited solution for electricity delivery and distribution as it faces increasing challenges in renewable resources, energy storage integration, and high asset costs. SG technology has emerged as an indispensable modernization instrument for enhancing present electrical systems in response to these limitations. By utilizing advanced network communication and monitoring technologies, SGs enable the efficient management of electricity transport from multiple generation sources to meet fluctuating end-user demand. Incorporating renewable energy sources and distributed generation (DG) constitutes a significant advancement in the power infrastructure. The nomenclatures used in this study are listed in Table 1.

Increased connectivity and reliance on technology also introduce new cyber threat vulnerabilities. Cybercriminals can use SG communication networks to launch large-scale attacks, including Denial of service (DoS), replay attacks (RA), time delay attacks (TDA), time synchronization attacks (TSA), false data injection attacks (FDIA), load redistribution attacks (LRA), Malicious command injection, and Malware attacks. The consequences of the mentioned CAs can be severe, ranging from economic losses to blackouts and disruptions to vital infrastructure. In addition, they can lead to the theft of sensitive data, such as customer and company information.

**Fig. 1:**
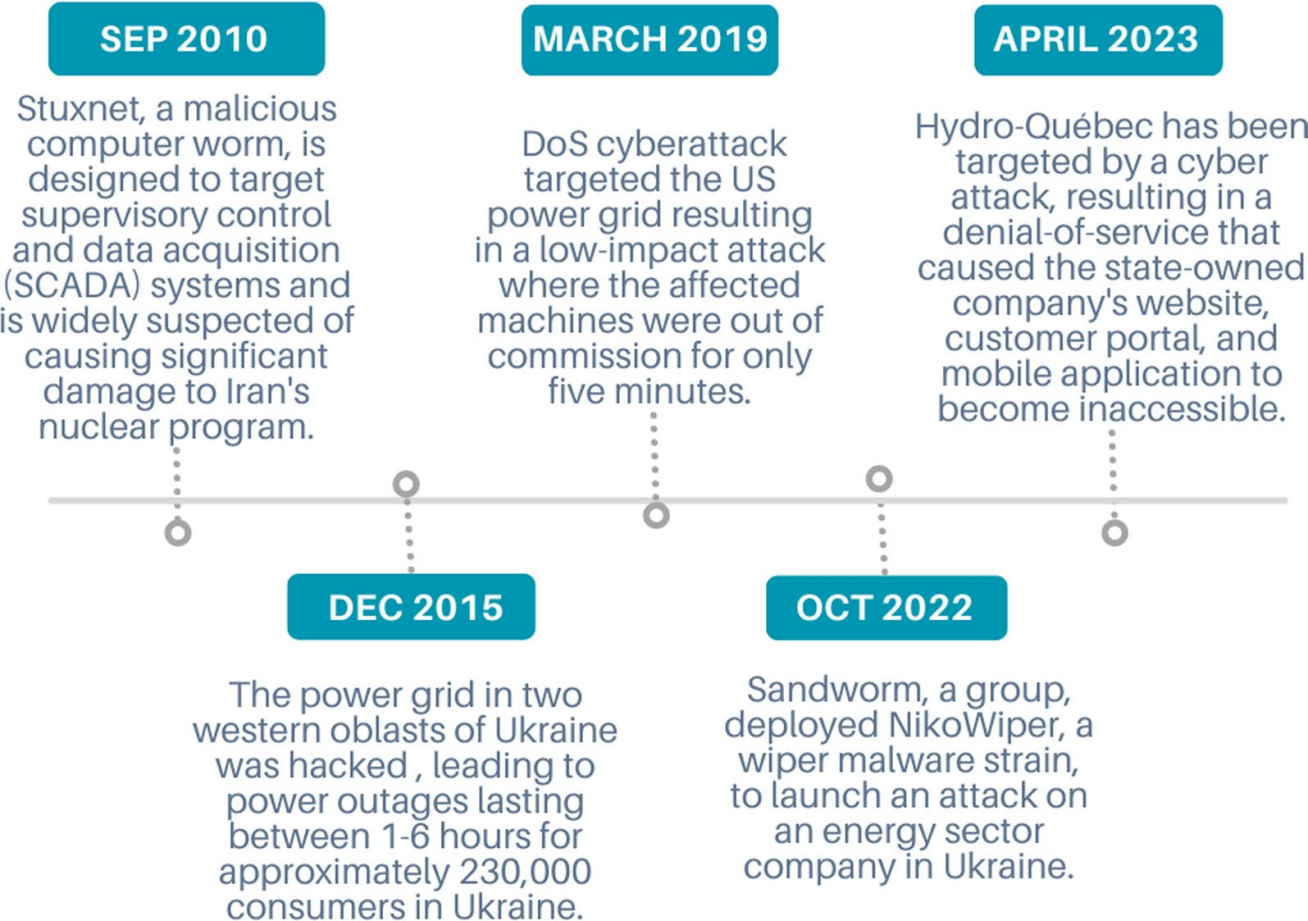
Timeline and history of industrial and energy-producing facilities cybersecurity attacks

As depicted in Fig. 1, a number of cyber incidents involving power systems demonstrate the importance of addressing cybersecurity concerns (Harper 2019; Lakshmanan 2023; Donghui Park 2017; Keizer 2010; Radio-Canada 2023). As cyber threats become more complex and prevalent, it is crucial to develop robust security measures to safeguard the interconnected power infrastructure. This paper aims to discuss the various dimensions of SG CAs and explore effective strategies to enhance its security.

**Table 1:** List of abbreviations used in this study

Abbreviation	Description	Abbreviation	Description
ACE	Area control error	AGC	Automated generation control
AI	Artificial intelligence	AMI	Advanced metering infrastructure
ARIMA	Autoregressive Integrated Moving Average	AVR	Automatic Voltage Regulator
BC	Blockchain	BCS	Binary Cuckoo Search
BDDE	Bad Data Detection And Elimination	BPSO	Binary Particle Swarm Optimization
CA	Cyber-Attacks	CIA	Confidentiality, Integrity, Availability
CITPF	Cyber-Informed Transmission Planning Framework	CISA	Cybersecurity and Infrastructure Security Agency
CIS	Consumer Information System	CR	Cognitive Radio
DC	Data Concentrator	DoS	Denial of Service
DDoS	Distributed Denial Of Service	DG	Distributed Generation
DMS	Distribution Management System	DNN	Deep Neural Network
DNP3	Distributed Network Protocol-3	D-Pmus	Distribution Phasor Measurement Units
DT	Decision Tree	ED	Economic Dispatch
ELM	Extreme Learning Machine	EMS	Energy Management System
ENN	Extended Nearest Neighbors	FDIA	False Data Injection Attacks
FS	Feature Selection	GA	Genetic Algorithm
HAN	Home Area Networks	HMI	Human-Machine Interface
HMM	Hidden Markov Model	IED	Intelligent Electronic Device
KF	Kalman Filter	KNN	K Nearest Neighbor
LFC	Load-Frequency Control	LMP	Locational Marginal Pricing
LRA	Load Redistribution Attacks	MDMS	Meters Data Management System
MiTM	Man-In-The-Middle	MLE	Maximum Lyapunov Exponent
MTUs	Master Terminal Units	NANs	Neighborhood Area Networks
Naspinet	The North American Synchro-Phasor Initiative Network	NESCOR	National Electric Sector Cybersecurity Organization Resource
NERC	North American Electric Reliability Corporation	NIST	National Institute Of Standards And Technology
NTP	Network Time Protocol	OMS	Outage Management System
Pevs	Plug-In Electric Vehicles	PLC	Power Line Communication
PLCs	Programmable Logic Controllers	PMU	Phasor Measurement Units
POMDP	Partially Observable Markov Decision Process	PTP	Precision Time Protocol
QDA	Q uadratic Discriminant Analysis	QoS	Quality of Service
RA	Replay Attacks	RF	Random Forest
RL	Reinforcement Learning	RSS	Received Signal Strength
RSSI	Received Signal Strength Indicator	RTU	Remote Terminal Unit
SCADA	Supervisory Control And Data Acquisition	SCMS	Smart Charging Management System
SE	State Estimate	SG	Smart Grid
SM	Smart Meter	TDA	Time Delay Attacks
TDOA	Time Difference of Arrival	UMAP	Uniform Manifold Approximation and Projection
WAN	Wide Area Networks	WSN	Wireless Sensor Networks

Several research papers have been published on the topic of cybersecurity in SGs, each with its own unique scope and areas of interest. Some papers focus on a particular part of the SG, such as Mohan et al. (2020), Saxena et al. (2021) and Chen et al. (2020) which all target load-frequency control (LFC). Other reviews concentrate on specific attack types, such as DoS attacks, as seen in Raja et al. (2022) and Ortega-Fernandez and Liberati (2023) or FDIA (Liang et al. 2016). Certain papers focus on the solution techniques, such as artificial intelligence (AI) (Omitaomu and Niu 2021; Ali and Choi 2020) and blockchain (BC) (Musleh et al. 2019; Alladi et al. 2019). However, several reviews cover different aspects of the field, including the architecture of SGs, various types of attacks, and solutions based on different techniques. These types of reviews provide a holistic view of cybersecurity in the SG. Table 2 provides a comparative analysis of these reviews. Year of publication, presentation of architecture and communication standards, SG cyber-physical attacks, and solutions presented in each reference are the primary aspects compared.

**Table 2:** Summary and comparison of surveys and reviews on attacks and countermeasures in SGs

References	Architecture and communication technologies	Attacks	Solutions
Kayastha et al. (2014)	Data communication network architecture and protocols	Vulnerabilities in sensor nodes, network devices, and protocols	Protection systems including encryption and data compression
Kabalci (2016)	Distributed communication architecture	General information and potential threats	Cybersecurity requirements
Kumar et al. (2019)	Smart metering infrastructure (SMI)	Threats in system-level security	Security and privacy requirements for on SMI
Al-kahtani and Karim (2019)	Some definitions related to securing SG systems	Common attacks classified based on type such as GPS spoofing, TSA, FDIA	Countermeasures classified based on types of attacks
Mohan et al. (2020)	General block diagram of multi-area LFC system	Identification of attack points, discussion of attack strategies	Brief review of existing detection and defense mechanisms against CAs on LFC
Zhang et al. (2021)	Not covered	Existing attacks classified based on target components	Defense approaches based on watermarking and data-driven approaches
Kawoosa and Prashar (2021)	Conceptual SG model	Evaluation of numerous existing attacks based on CIA principle	Background of BC and IoT-based security solutions
Abdelmalak et al. (2022)	Summary of Cyber-Physical Power System (CPPS) layers and dependencies among system layers CPPS Modeling Methods	Not covered	Not covered
Khoei et al. (2022)	Overview of SG Infrastructure (architecture, protocols, and standards)	Classification of attacks based on the OSI model	Detection techniques classified based on used technologies
Nafees et al. (2023)	Introduction of devices and systems that exist in the SG without communication technologies	Exploration of characteristics of CAs using MITRE ATTACK and cyber kill chain threat modeling approach	Detection and monitoring techniques and tools categorized based on detection technique and IDS deployments

The previous surveys have their own advantages. Some of them include the SG’s architecture and its communication standards (Khoei et al. 2022). However, the coverage area is limited because they do not involve the connection between all SG devices and systems. In this study, we demonstrate the most prevalent SG devices and systems, as well as their interdependencies. This type of holistic architectural diagram enables a deeper understanding and identification of key pathways through which cyber threats can propagate, leading to better comprehending the potential impact of attacks on the system and related devices. Additionally, it allows for a systematic risk assessment of the SG’s cybersecurity. Through analyzing the connections between elements, cybersecurity professionals are better equipped to identify vulnerabilities, weak points, and attack vectors facing the network. From an architectural perspective, a detailed view of device interconnectivity allows for the identification of key components, their roles, and their interdependencies within the SG. It helps in designing robust communication protocols, data management systems, and control mechanisms to facilitate seamless integration and interoperability among devices. In terms of CAs, the preponderance of previous research provides only one classification for each one. It varies across different papers, with some based on the target points on the architecture (Zhang et al. 2021), others on types of attacks or threats (Al-kahtani and Karim 2019), and yet others on the Confidentiality, Integrity, Availability (CIA) principle (Kawoosa and Prashar 2021) or Open Systems Interconnection (OSI) model (Khoei et al. 2022). However, our paper classifies CAs based on two criteria: first, their types, and then their target devices and domain.

On the other hand, previous papers typically focus only on protection and detection mechanisms in their solutions sections (Kayastha et al. 2014; Khoei et al. 2022; Nafees et al. 2023). In our study we also include identification methods, as well as response and recovery mechanisms for the system after an attack, taking into account the NIST framework. Many organizations from the sectors of energy, transport, banking, health, water, and digital and financial market infrastructures consider the NIST cybersecurity framework in order to assure a reasonable level of cybersecurity. As a result, our study differed from the other reviews by covering all the steps of assuring security and not only concentrating on specific steps, like detection and protection. We provide a list of recent scientific research works in the identification of risk, response, and recovery function. This paper supports organizations seeking to follow a complete security routine by taking into consideration the scientific countermeasures at each step. Additionally, NIST explicitly states that no function is more important than another and calls for a balance of the five functions. This balance is not highlighted in previous literature. Our study sheds light on the existing gaps in scientific research concerning the various functions of the framework. Specifically, we identify a need for more extensive research in the areas of response and recovery mechanisms.

The article’s main contributions are as follows:

- Presenting a comprehensive architecture of the SG, accompanied by a diagram of the electrical network, displaying the various devices and key components so that the connections between these elements can be visualized more clearly.
- Grouping the various communication technologies based on the SG communication Networks: Home Area Networks (HANs), Neighborhood Area Networks (NANs), and Wide Area Networks (WANs).
- Providing a list of CAs in the SG, classified by type and target points.
- Categorizing cybersecurity solutions and research according to the NIST Cybersecurity Framework, which allows organizations to gain a better comprehension of the specific areas of cybersecurity. These include identifying threats, protecting against them, detecting attacks, responding to cyber incidents, and recovering systems and data after a CA.
- In the assault prevention approaches, BC-based solutions are prioritized in the discussion, while AI-based strategies are investigated as the primary components of the presented detection mechanism.
- Providing open research issues and future trends that must be considered in the future.

The structure of this paper is as follows: In Section 2, an overview of the SG system and its architectures, networks, and technologies is presented. This section also includes information about the components and communication links between them. Section 3 provides a classification of CAs that target each component of the SG and reviews them based on categories. In Section 4, existing countermeasures against various CAs are summarized. Section 5 outlines several research challenges and future research directions. Finally, the paper concludes with a summary of the research in Section 6.

## Overview

### Architecture of the SG

**Fig. 2:**
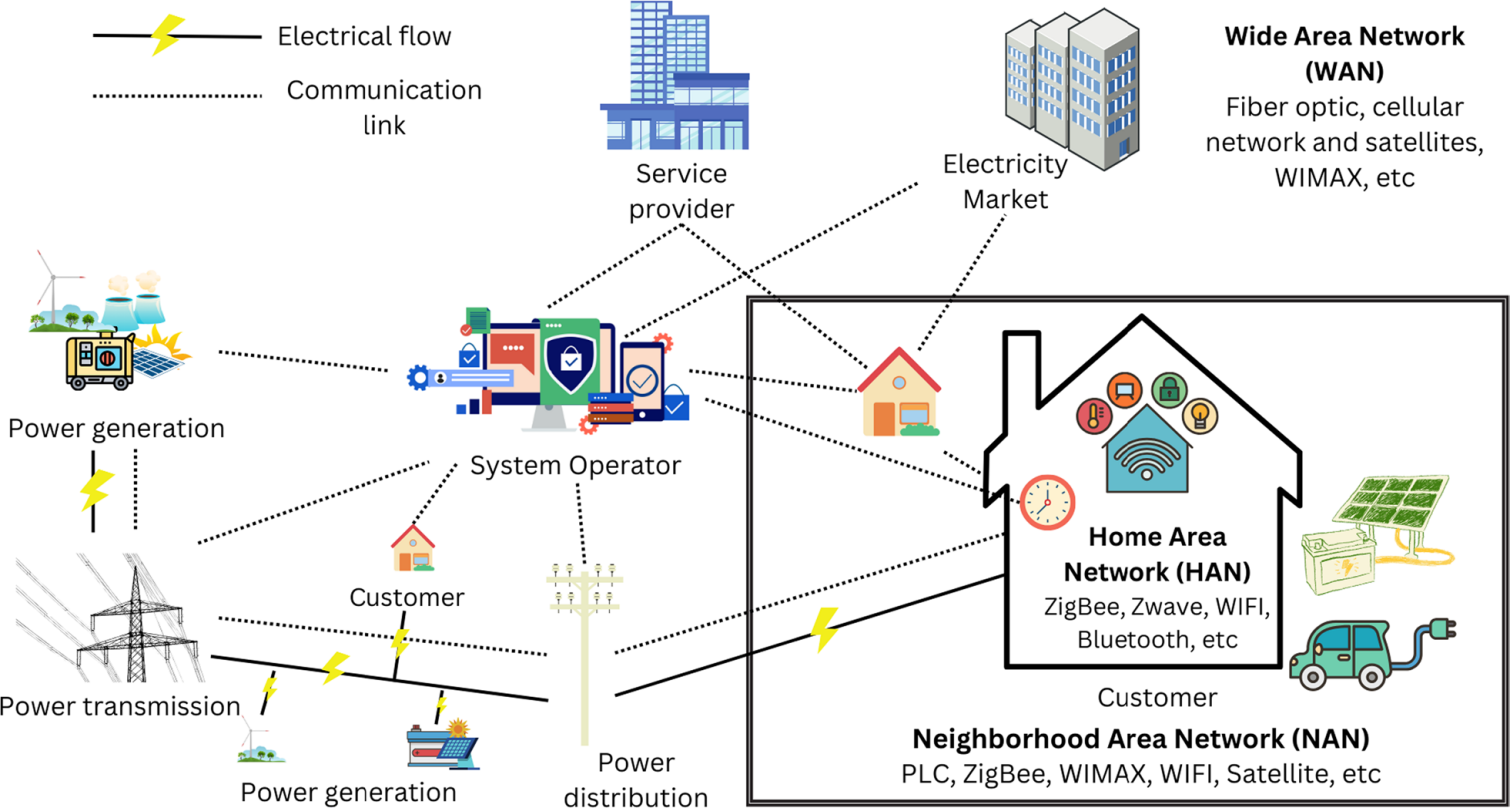
Smart grid architecture

The architecture of the SG is a subject of ongoing research and study by various stakeholders, including researchers, service companies, and electricity producers. Several models have been proposed, each with its unique features and characteristics. In this context, the authors in Ananthavijayan et al. (2019) review several standard architectures. One example is the North American Synchro-Phasor Initiative Network (NASPInet) (Gorton et al. 2012). Another example of a SG architecture model is the Grid Operation and Planning Technology Integrated Capabilities Suite (GridOPTICS) (Bobba et al. 2010). The NIST has also developed a SG architecture model (Standards 2021), which includes seven logical domains: customer, markets, service provider, operations, transmission, distribution, and bulk generation as shown in Fig. 2.

#### Customer

This is the domain where power is used, but it is becoming increasingly actively managed and generated. It includes appliances, entertainment systems, lighting systems, and energy storage and generation (solar, wind, etc.). Sensors in the customer domain enable clients to manage and monitor their energy consumption and generation. A smart meter (SM) that measures customer’s energy consumption is used for the smart measurement. Thus, SMs comprise metering and bidirectional data flow communication infrastructures. The communication component of an SM consists of network connection and control infrastructure, allowing the meter to interact with remote centers and execute control commands. SM transfers the measured data through a gateway to data concentrators (DC) (Kabalci 2016; Kazičková and Buhnova 2016). The communication between companies and SMs enables demand response (DR) programs from both consumer and utility sides in specific scenarios. DR allows end-users to track their energy consumption and production, adjust habits to off-peak hours, and participate in dynamic pricing programs. It can also be integrated into home energy management systems for automated appliance control (Abrahamsen et al. 2021; Siano 2014).

#### Markets

The market is a crucial component of the SG architecture. It serves as a platform where grid assets and services, including electricity, are purchased and traded.

#### Service provider

The Service Provider is an interconnected component of the SG architecture. Actors in the Service Provider domain perform services to assist the business activities of power system producers, distributors, and customers, such as billing and customer account management.

#### Operating and monitoring systems

Several active systems are utilized to assess and efficiently operate the power system. By monitoring and controlling the customers’ consumption and the flow of electricity, managing energy storage resources, and responding to emergencies or disruptions, the Operations domain helps to ensure the stability and resilience of the electricity grid. It is composed of several components, each of which plays a critical role in the efficient and reliable operation of the electricity system. For instance, Supervisory Control and Data Acquisition (SCADA), is the control system responsible for monitoring, measuring, and interpreting real-time data from the electrical power grid. This system is composed of several components, including sensors, control devices, and a central computer system. The Human–Machine Interface (HMI) provides a program interface between SCADA hardware and software components (Yadav and Paul 2021). The SCADA system is succeeded by an automated generation control (AGC) system, which maintains the balance between the electrical load and generation. It controls the output of power generation units to ensure this balance. Additionally, the Operations domain includes the energy management system (EMS) which manages and optimizes the operation of the power system.

#### Transmission power

Transmission is the bulk transfer of electrical power from generation sources to distribution through several substations. Typically, the transmission network is monitored and controlled by the SCADA system. It employs a communication network, field monitoring devices, and control devices such as Remote Terminal Unit (RTU) that collects real-time data and information from sensors connected to the physical environment, substation meters, protection relays, power quality monitors, and Phasor measurement units (PMU). The PMUs are used to measure the direction and amount of power flow based on phasor measurements that are based on the magnitude and phase angle of voltage, and current. Additionally, it contains Intelligent Electronic Devices (IED) which provide control and automation functions, and protect power systems in the SG.

#### Distribution power

Electricity distribution is the final step in delivering power to end users. The distribution domain transports power from the transmission system to consumers. To monitor the distribution network, advanced monitoring systems such as Advanced Metering Infrastructure (AMI) are used. AMI is an integrated system of SMs that collect, measure, and analyze energy usage data, with the help of specialized software, hardware, communication networks, and customer-associated systems (Yan et al. 2011). The metering data obtained from the electricity consumption of home appliances is forwarded to the meters data management system (MDMS). MDMS is responsible for data storage management and data analysis. Distributed MDMSs can be deployed close to SM, with each MDMS responsible for storing and processing data from SM in the near area via several concentrators (Zhou et al. 2012). This system enables the utility company to have real-time insights into energy consumption. Additionally, the Distribution domain utilizes RTUs, Distribution Phasor Measurement Units (D-PMUs), and IEDs to monitor, control, and optimize the operation of the distribution network. D-PMU is the designation for PMUs developed specifically for distribution systems (Liu et al. 2020). These devices are critical for improving the reliability and efficiency of the electricity supply and enabling the transition to a more sustainable energy system.

#### Generation power

This area contains a wide range of primary energy resources and technologies, such as chemical combustion and nuclear fission, as well as hydro, wind, solar, and geothermal. Thus, intelligent power generation should be linked to demand forecasting and AGC to adjust the power output of generators in response to load variations for ensuring frequency control (Kabalci 2016).

### SG communication networks

SGs are made up of three different types of networks: HANs, NANs, and WANs, Fig. 2. HANs are designed to connect and control devices within a home, NANs are intended to cover a neighborhood or a small geographical area, and WANs are responsible for managing the entire grid. To facilitate communication between these different networks, various wired and wireless communication technologies are used. Wired communication technologies include fiber optic, power line communication (PLC), and Ethernet, while wireless communication technologies include Z-Wave, Bluetooth, ZigBee, WiFi, WiMAX, wireless mesh, cellular network, and satellite. Each technology has its advantages and disadvantages and is used in different parts of the SG. Table 3 provides an overview of the technologies used in the SG, where they are used, their data rate, coverage range, advantages, and disadvantages (Abrahamsen et al. 2021; Lotha 2023; BasuMallick 2022; Electronics 2023).

**Table 3:** Technologies used in the smart grid

Type	Technology	Data rate	Coverage range	Advantages	Disadvantages	Network
Wireless connection	Bluetooth	Up to 1–3 Mbps	10–30 m	Low cost, Low power consumption, Widely available	Limited coverage range, Vulnerable to interference, Inadequate security	HAN
	ZigBee	40–250 kbps	10–100 m	Low cost, Low power consumption, Easy to install and maintain	Low bandwidth, Inadequate security	HAN, NAN
	Z-Wave	9.6–100 kbps	Up to 100 m	Easy to install and maintain, Low power consumption	Limited coverage range, Low bandwidth, Limited data rate	HAN
	WiFi	2 Mbps–1.7 Gbps	Up to 100 m	High data rate, High flexibility, widely available	Vulnerable to interference, High power consumption	HAN, NAN
	WiMAX	75 Mbps	50 km	Large coverage area, High data rate	Connection problems in bad weather, High installation cost	NAN, WAN
	Cellular network	Up to 20 Gbps	100 km	High data rate, Widely available, Large coverage area	Vulnerable to congestion	WAN
Wired connection	Ethernet	Up to 10 Mbps–400 Gbps	Up to 100 m	High data rate, Low latency, Reliable	Limited coverage range	HAN, NAN
	PLC	10–500 Kbps (NB-PLC)	Up to 3 km (NB-PLC)	Low installation cost, Available infrastructure	Susceptible to noise and interference, Complex routing, Limited range	HAN, NAN
		Up to 300 Mbps (BB-PLC)	Up to 1.5 km (BB-PLC)			
	Fiber optic	Up to 100 Gbps	Up to 100 km	High data rat, Low signal loss, High reliability, and security	High installation cost	WAN

#### Home area network

A HAN in the context of SGs refers to a network that connects smart devices within a home to the power grid. The main purpose of a HAN is to enable communication and information sharing between smart devices such as SMs, thermostats, appliances, and electric vehicles. It typically uses wired and wireless technologies such as Ethernet, Wi-Fi, Zigbee, and Bluetooth to enable communication between devices in order to provide real-time energy consumption data, DR, and home automation services. HAN can also communicate using PLC, which utilizes existing wireline connections to transmit data from one node to another. The PLC runs at two different data rates known as narrow-band PLC (NB-PLC) and broadband PLC (BB-PLC) (Kabalci 2016; Kumar et al. 2019; Colak et al. 2021a, b; Gungor et al. 2011).

#### Neighborhood area networks

A NAN is a grouping of many HANs. Several viable technologies, such as PLC, WiMAX, Zigbee, WIFI, cellular networks, and wireless mesh networks, have been widely used in NAN to offer communication. In the wireless mesh network, each smart mesh meter collects its data and acts as a router for other SMs to transmit consumption usage information to the DC. Via unlicensed radio, a mesh network can operate up to 900 MHz. The Internet is utilized to connect the smart metering mesh network to the distributed DCs, which are typically placed a few kilometers away (Kabalci 2016; Kumar et al. 2019; Colak et al. 2021a, b; Gungor et al. 2011).

#### Wide area network

The WAN is the primary network that can serve to connect extensively dispersed smaller networks for power systems in different areas. This high-bandwidth link network is capable of long-distance data transmission for sophisticated monitoring and sensing applications. WAN enables bidirectional connection for SG system automation, monitoring, and communication.

For high-speed communications, optical fiber connections are utilized as usual. When a fiber optic is deployed in networks in the overhead transmission and distribution domains, various communication services can be provided to power utilities for their purposes (e.g., system protection, load, and DG management, distribution automation, diagnostic monitoring) (Lazaropoulos and Leligou 2022). Although wired connection approaches are chosen in several automation systems, wireless communication techniques have also received considerable interest. Specifically, the cellular network, WiMax, and satellites offer efficient Internet connectivity for automation and metering equipment (Kabalci 2016; Colak et al. 2021a; Gungor et al. 2011). Wireless Sensor Networks (WSN) represent a new frontier in wireless communications for SGs, particularly for metering applications. Cognitive radio (CR) approaches can improve the effectiveness and usage of a radio frequency spectrum in wireless networks. With CR, the spectrum allocated to licensed users (i.e., primary users) can be accessed selectively and dynamically by unlicensed users (i.e., secondary users). In Qiu et al. (2011), the application of CR network in SG is investigated.

### The components and communication links of the SG

**Fig. 3:**
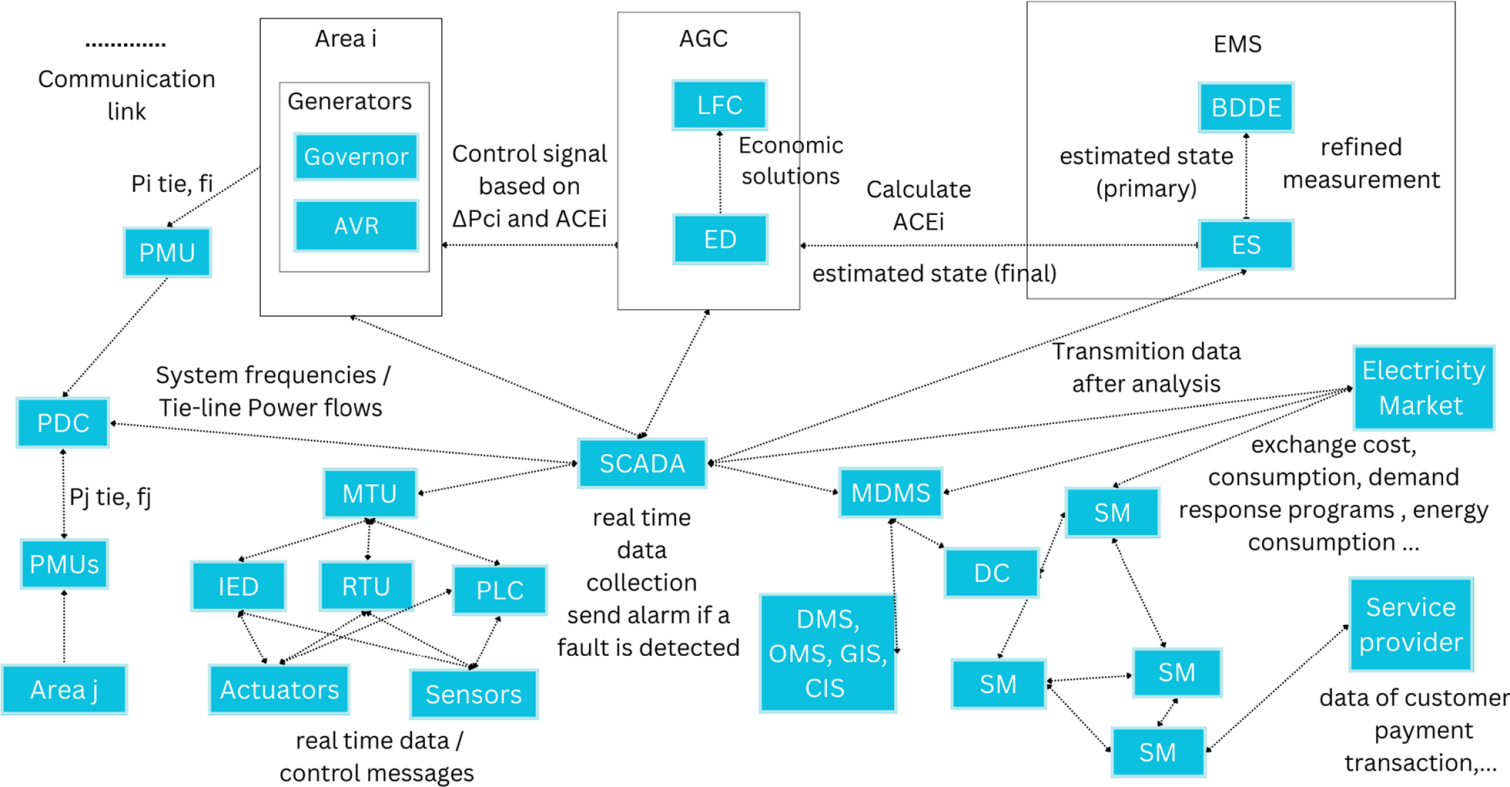
Diagram illustrating the components of the smart grid and the communication links

The SG is a complex system that integrates various components, relying on advanced communication to manage and control them effectively. In this section, we present a diagram in Fig. 3, illustrating the components of the SG and the communication links between them, emphasizing the critical role of each component. This diagram offers a comprehensive overview of the SG’s structure and functionality.

By continuously monitoring critical parameters, the control system can promptly detect abnormalities or faults. This is achieved through RTUs that link physical objects to the automation system, transmitting telemetry data and controlling connected objects based on received messages (Zhang 2010). Programmable Logic Controllers (PLCs) and IEDs are also used to interface with sensors and actuators through input and output modules (Lisowiec and Nowakowski 2013). Then, the real-time data collected from sensors, are forwarded to Master Terminal Units (MTUs), which serves as the first central monitoring station (Yadav and Paul 2021).

On the other hand, besides monitoring power system quality, the SG incorporates other functionalities like dynamic pricing, DR, outage notification, power connect/disconnect, and theft detection, executed through SMs. These SM measure, store, display, and transmit energy usage data to utility companies using two-way communication (wireless/wire-line) and act as home gateways that collect energy consumption readings, send them to control data centers, and execute control commands received from the utility (Kumar et al. 2019). The MDMS controls the meter’s current configuration and connects to the AMI headend device, which aggregates collected data (Sridhar et al. 2011). For that, MDMS comprises an Outage Management System (OMS), Geographic Information System (GIS), Consumer Information System (CIS), and Distribution Management System (DMS). The OMS enables MDMS to detect abnormal situations and intervene quickly, and DMS collaborates to manage communication and management systems. The GIS and CIS systems collect data such as utility location, consumption rates, and billing information about SM and consumers (Kabalci 2016). Interactions between SMs and the utility center, and between SMs and the electric market, involve a multi-hop and hierarchical communication network (Wang and Lu 2013). As described by Kumar et al. (2019), every smart mesh meter collects its data and serves as a router for other SMs to send their consumption usage data to the DC.

Following that, addressing the controller, it is important to note that power systems are typically divided into control regions, each monitored and controlled by a separate SCADA system (Vrakopoulou et al. 2015). SCADA gathers information about an electric system, particularly system frequency, generator outputs, and actual interchange between the system and adjacent systems (Zhe et al. 2021). After gathering measurements in the control center, state estimation is conducted by the EMS to determine the most probable system state, considering measurements might be inaccurate or incomplete (Vrakopoulou et al. 2015). The process estimates state even when field devices provide imperfect measurements or the control center fails to receive measurements due to device or communication channel malfunction (Sridhar et al. 2011). Additionally, the EMS provides the bad data detection and elimination (BDDE) process, that removes bad data from the measurements (He and Yan 2016). Based on the estimated state, the SCADA system alerts the operator if control actions should be taken (Vrakopoulou et al. 2015).

Moreover, balancing and frequency control occur across a continuum of time utilizing diverse resources at multiple steps (Zhe et al. 2021). First, the primary control units detect frequency/speed changes of generator units using a sensor and adjust governor and turbine settings to maintain the voltage at a specified set point (Mohan et al. 2020). It is provided by Automatic Voltage Regulator (AVR) (Sridhar et al. 2011). Then, the AGC is a secondary control loop that corrects inter-area tie-line flow and frequency deviation (Sridhar et al. 2011) and restores frequency to its scheduled value, usually 60 Hz (Zhe et al. 2021). Unlike governor control, secondary control schemes allow frequency control of multiple generators operating in parallel, sharing large electrical loads (Mohan et al. 2020). Finally, Tertiary control follows secondary control to guarantee sufficient secondary control reserve through manual or automatic change of generator or participating load working points (Mohan et al. 2020). In our review, we focus on the vulnerability of the AGC unit to cyber attacks, specifically FDIA, considering its crucial role in ensuring a constant frequency and preventing cascading failures or blackouts.

An AGC system is comprised of two primary functions, the LFC, which maintains the load-generation power balance and system frequency; and the Economic Dispatch (ED), which distributes the generation among generators with minimal operating costs (He and Yan 2016). First, the LFC incorporates an Area Control Error (ACE) into the frequency feedback loop. ACE values for each area are calculated using frequency and tie-line power flow measurements received from PMUs (Mohan et al. 2020; Kabalci 2016).

The $ACE_i$ is the linear combination of the frequency deviation of area i and the tie-line power deviation between area i and other areas as expressed by equation 1.

1 $$\begin{aligned} ACE_i = \beta \delta f_i + \sum _{j=1,j\ne i}^n \delta Ptie,ij \end{aligned}$$

Where $\delta f_i$ is the area $i$ frequency deviation and $\delta Ptie,ij$ is the area $i$ and area $j$ tie-line power deviation. The LFC calculates the required power deviation $\delta Pci$ (Li et al. 2020). Then, the active output power of the generators is adjusted to maintain a dynamic equilibrium between the active output power of the area and the demand (Li et al. 2020).

As mentioned previously, AGC also supports an ED function that interacts with the LFC function to reschedule the entire system’s generation and mitigate generation costs relative to system-wide performance (Li et al. 2015). To accomplish this, measurements are sent to the ED, and then a feedback signal that regulates the generated power is sent back to the AGC-participating generators via the SCADA system (Vrakopoulou et al. 2015). The magnitude of the control command sent by AGC to the local generation units for each controlled generator is proportional to the coefficient updated by the ED algorithm (Huang et al. 2018).

## Cyber attacks in SG

Modern technologies and complex networks, have made the power infrastructure increasingly vulnerable to CAs. Understanding the sorts of cyber assaults that may be conducted against the grid and their entry points is necessary to protect it. This section has two parts. The first classifies attacks by category. Grouping SG attacks by type is essential for designing focused defenses against specific threats. Man-in-the-middle (MiTM) attacks, RA, TDA, FDIA, LRA, DoS, TSA, and malicious command injection and malware attacks are covered in this section. Each category’s special characteristics and possible effects are analyzed. However, the second part classifies attacks by target points. Cyber attackers can target SG components with particular weaknesses. It discusses potential cyber assaults on each component, including customers, power markets and service providers, SCADA systems and WAN communication technologies, measurement devices, and AGC systems.

### Types of cyber attacks

Each type of attack has unique characteristics and potential consequences. For instance, MiTM attacks involve intercepting communication between two devices, while RA and TDAs manipulate historical measurement data or control signals. TSA and FDIA target timing information and exploit vulnerabilities in bad data detection techniques to manipulate meter measurements and influence state estimation, respectively. On the other hand, DoS attacks aim to render messages inaccessible to the intended destination. Identifying these attack categories, specific countermeasures can be developed to protect against them and can help in prioritizing cybersecurity efforts and resources, as some attacks can have more severe consequences than others. Therefore, this section provides an overview of each of these attack categories and examines their potential impact on a SG.

#### Man-in-middle attack (MiTM)

MiTM attacks pose a variety of threats to a SG. In a MiTM attack, an attacker embeds himself within a dialogue between two devices to either eavesdrop or spoof one of the devices, making the passage of information appear regular (Conti et al. 2016). Kulkarni et al. (2020) examines the potential security dangers posed by a MiTM attack on a power system while focusing on the weaknesses in the Modbus TCP/IP protocol used for communications. The authors in Fritz et al. (2019), present a prototype of a MiTM attack to be implemented on a SG emulation platform. They offer a method for breaking the integrity and authenticity of IEEE Synchrophasor Protocol packets. The physical distance between PMUs and the Phasor Data Concentrator (PDC) makes it harder to detect packet interception and the speed at which the PDC must acquire data provides minimal time for encryption, authentication, and integrity checks.

#### Replay attack (RA) and time delay attack (TDA)

Getting the control signal at the appropriate moment is essential for controlling the system. The TDA affects the system by randomly delaying the transmission and reception of packets (Wu et al. 2019). The RA strategy is implemented by recording sensor measurements for a certain time window and replacing actual sensor measurements leading to modifying control signals, or by maliciously repeating the control signals sent from the operator to the actuator (Zhu and Martinez 2013). Hence, in both types, the control center drives the system states out of their normal values by using historical measurement data or control signals, which could render the power system damaged (Mo and Sinopoli 2009).

#### False data injection attack (FDIA)

State estimation is the technique of estimating unobserved state variables in a power system based on meter readings. FDIA were introduced in Liu et al. (2011) to manipulate meter measurements and covertly influence the outcome of state estimate (SE) by exploiting the vulnerabilities of bad data detection techniques in the EMS. The attacker injects a vector of observed measurements that may contain malicious data that cannot be detected by BDDE. As many power system applications (such as ED that meets the expected system demand at the lowest cost possible) rely on the results of state estimate, faked estimation may confuse the system operation and control functions and lead to wrong decisions (Liang et al. 2016). In addition, FDIA affects stability. The authors of Chen et al. (2016) demonstrated how an FDIA attack might lead to unnecessary rescheduling of generation and load shedding.

#### Load redistribution attack (LRA)

The authors of Yuan et al. (2011) introduced the LRA, a specific kind of FDIA, which can disrupt the power grid functioning by targeting the ED. The purpose of ED is to reduce the entire system operation cost (generation cost, load shedding cost, etc.) by re-dispatching the generated outputs. After the estimated state has been modified by an LRA, the false ED solution has the potential to force the system into an uneconomic operational state. There are two approaches for LRA: immediate and delayed attacking objectives. The immediate attacking objective is to maximize the cost of power system operations immediately following the attack, whereas the delayed attacking objective is to gradually overload the power lines, which can result in physical damage to the power system.

#### Denial of service attack (DoS)

A variety of measuring equipment, such as SMs, smart appliances, data aggregators, PMUs, RTUs, IEDs, and PLCs, are present in SGs. On these devices, several DoS targeting vulnerabilities are exploitable. In a power system, a DoS is an action that renders measuring device data inaccessible to the intended user, or prevents control commands from reaching actuators, and eventually causes system instability. This interrupt the operation of the SG since it is unable to log any events occurring at that time. A DoS attack consists of either flooding to overload a device or channel with data, or the manipulation of protocol and system weaknesses and abnormalities (e.g. jamming and routing attacks, etc) (Jhaveri et al. 2012; Liang et al. 2016; Xu et al. 2006). A puppet attack is a novel DoS attack that can result in preventing communication in an AMI network, as described by Yi et al. (2016). The intruder can designate any regular node as a puppet node and transmit attack packets to it. When the puppet node gets these attack packets, this node can be controlled by the attacker and can overflow the network communication capacity and node’s energy with additional packets. Instead of initiating an assault from a single source, another derivative attack from DoS in the power system is distributed DoS (DDoS) attacks launched concurrently from many dispersed systems (Raja et al. 2022).

#### Time synchronization attacks (TSA)

TSA is possible CAs on SG that target timing information. Some applications in SG require synchronous measurements, and the majority of measurement devices, such as PMU, may use GPS as a time source and Network Time Protocol (NTP) as a means of time distribution (Singh et al. 2015). In the case of GPS, the device synchronizes itself to the time reference received from a group of GPS satellites, while in NTP, the equipment clock operates as a slave device and adjusts its time to a reference received from a master clock device, which is equipped with an accurate clock. The most common protocol for NTP is the Precision Time Protocol (PTP). Both systems have been demonstrated to be susceptible to TSAs through GPS jamming, spoofing, and software compromise (Jiang et al. 2013; Zhang et al. 2013).

#### Switching attacks (SA)

A switching attack (SA) is a type of CA where the attacker discovers a switching sequence for the circuit breaker that induces instability in the phase angle (also known as the rotor angle) and frequency of the generator, compelling it to disconnect (Liberati et al. 2021). Coordinated SAs were proposed in Liu et al. (2011a, 2011b) where the transmission system is represented as a single-machine infinite bus system. This model includes a generator and a load linked to the bus through a breaker. In practical scenarios, a substation SA refers to the deliberate disconnection of various power equipment components, such as transformers, transmission lines, and buses, which are linked to substations via compromised local networks. This type of attack, by disconnecting the compromised substations, has the potential to introduce grid congestion, giving rise to various forms of instability stemming from subsequent events (Yamashita et al. 2020a). In response to the recommendations provided by the North American Electric Reliability Corporation (NERC) regarding the implementation of anomaly detection features in IP-based substations, the research (Yamashita et al. 2020a) focuses specifically on IP-based substations and indicates that SA through intrusion into an IP-based substations network can be possibly executed through a local computer that has complete access to all breakers and controlled switchgear in the substation, or through a digital relay that has partial switching control of the circuit breakers in the substation. Moreover, Yamashita et al. (2020b) discusses how switching attacks that involve the opening of circuit breakers at substations can be potentially carried out through direct IP-based IEDs widely used in the SG. Furthermore, switching attacks have the potential to trigger cascading tripping events, resulting in the occurrence of blackouts (Yamashita et al. 2020a).

#### Malicious command injection and malware attacks

Popping the HMI is one of the disruptive CAs aimed against the SG that may be used to introduce malicious commands. In this instance, an attacker exploits vulnerabilities in the device’s software or operating system and installs a remote shell, which enables the adversary to connect remotely to the server from the adversary’s computer. The objective of this attack is to gain unauthorized access and control over the compromised system. By mapping harmful commands, Lin et al. (2018) examines the effects of control-related attacks on the dynamic reactions of a power system.

CAs targeting SG systems include further malware attacks, such as logic bomb and Trojan horse. A logic bomb is an attack that is designed to execute a specific action when certain conditions are met and can result in system failure, auto-deletion of hard drives, data modification, etc. Dusane and Pavithra (2020). Trojan horse typically hides malicious malware as a nice software that the user is ready to execute (Namanya et al. 2018). Botnet (Liu et al. 2009), is a network of hacked remote control computers used to transmit malware, spam, and steal communications. Typically, botnets are installed covertly on the target computer, allowing an unauthorized person to remotely manipulate the target system for malicious purposes.

### Attack classification based on target points

**Fig. 4:**
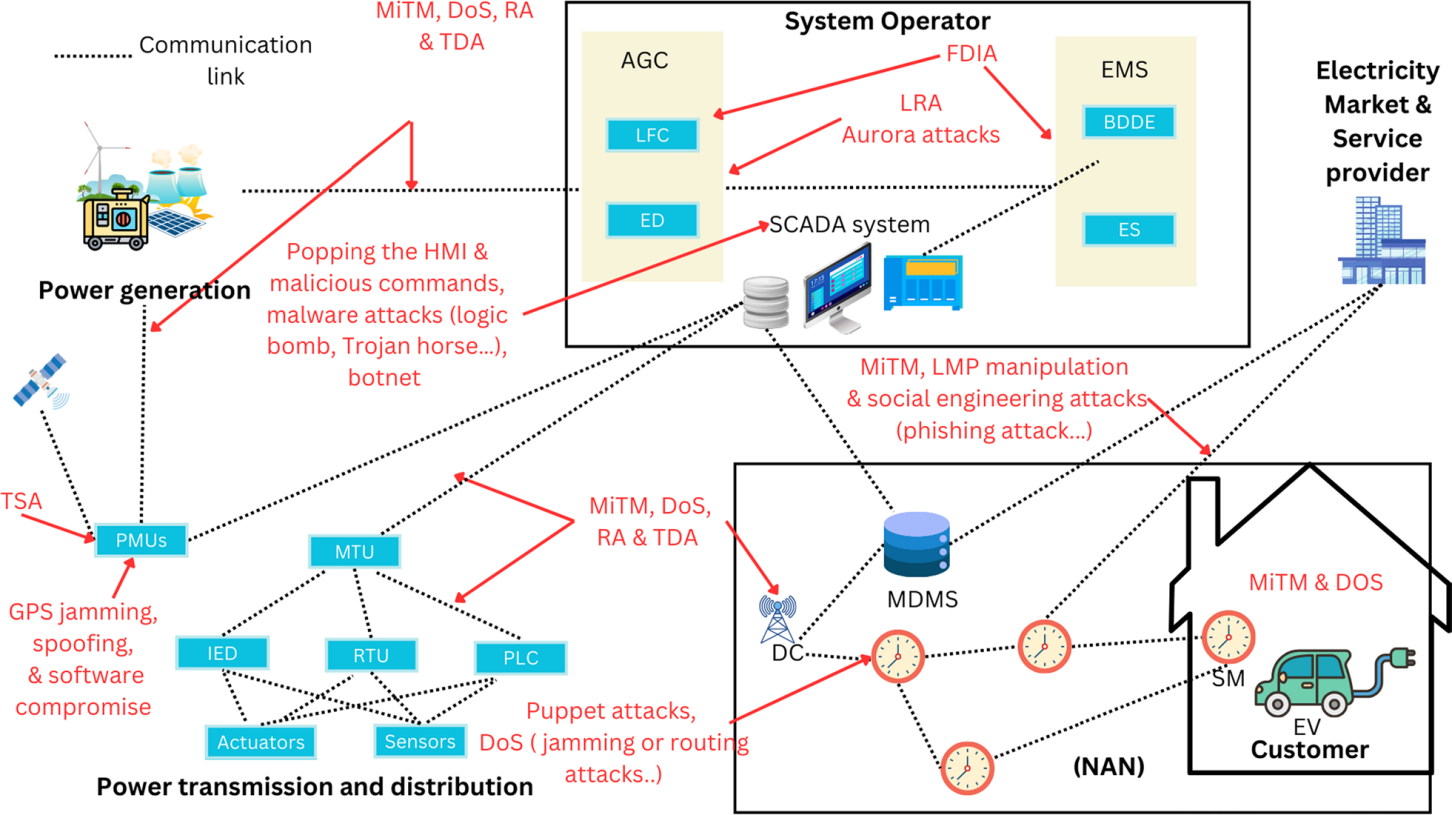
Attack points in the smart grid

One critical aspect of protecting the SG is identifying potential points of access for cyber attackers. In this context, attack classification based on target points is an essential tool for assessing the vulnerability of the SG. Various components of the SG can be targeted by cyber attackers, such as measuring devices, AGC, SCADA systems, communication technologies in WAN, the power markets, the service providers, and the customer as shown in Fig. 4. Each of these components has unique vulnerabilities that can be exploited by attackers, which can lead to severe consequences for the power system. This section provides an overview of the potential CAs that could be launched against each component.

#### Customer

To build a network, the equipment in the customer domain used widely wireless technology for information exchange between entities. As demonstrated in Lounis and Zulkernine (2020) wireless networks are vulnerable to DoS, MiTM, and spoofing attacks. An attacker may be able to intercept wireless communications if unauthorized eavesdropping occurs on the communication channels or compromise vulnerable meters and arbitrarily alter their readings. Through intercepting, an adversary can obtain information, such as a consumer’s power consumption, and deduce the consumer’s daily routine, and personal information and pose grave risks to the privacy of customers. This domain also consists of a smart charging management system (SCMS) that optimizes the charging of plug-in electric vehicles (PEVs) and offers various grid services. The article (Bhusal et al. 2021) addresses the notion of SCMS and offers a full examination of cybersecurity elements related to it, such as MiTM attacks, data intrigued attacks, and denial of charging, in addition to their potential effects on the power system.

Furthermore, integrating renewable energy sources (RES) and Distributed Energy Resources (DERs) on the customer side, where power outputs can be inconstant, smart inverters play a crucial role in addressing these challenges. However, it is worth noting that they also introduce supplementary cybersecurity vulnerabilities, as emphasized in prior scientific research (Ustun 2019; Li and Yan 2022). The deployment of several smart inverters at client locations broadens the potential targets for attacks and enhances their accessibility, particularly when integrated with building automation systems and other public information technology networks (Qi et al. 2016). This connectivity, frequently supported by third parties like smart inverter makers and DER aggregators, enables remote access to monitor, configure, and manage smart inverters. The presence of remote access capabilities creates vulnerable routes that may be exploited by malicious actors for the purpose of remote code injection and execution, which may result in significant repercussions. For instance, unauthorized access has the potential to cause blackouts by the disconnection or reduction of a substantial amount of solar power, particularly during sunny days. An illustrative example of the vulnerabilities in smart inverters is demonstrated in experiments targeting SunSpec Modbus-based inverters (Onunkwo et al. 2019). The conducted studies unveiled the possibility of conducting packet replay attacks, which allow for the interception, alteration, and subsequent retransmission of packets carrying phase voltages, DC voltages, current, and power data from the inverter to an external device, utilizing tools such as Netcat. To address these cybersecurity concerns, extensive efforts have been undertaken in the power industry (Li and Yan 2022). The National Electric Sector Cybersecurity Organization Resource (NESCOR) has provided guidance on the architecture and cybersecurity requirements specific to DERs ((NESCOR) 2015).

#### Power markets and service providers

The authors of Jia et al. (2013) presented a comprehensive analysis of the impact of Data integrity attacks on the energy market, especially the locational marginal pricing (LMP). As LMP highly depends on the correct topology and exact real-time measurements, any errors in these vectors have a significant effect on LMP. A study that exploits the economic impact of FDIA, on electric power market operations is detailed in Xie et al. (2011) and concludes that manipulating the data used to calculate electricity prices in the market by attackers can lead to significant financial losses and reduce trust in the power market, which may result in a decrease in the number of market participants. In addition, companies and customers are targeted and affected by social engineering attacks (Salahdine and Kaabouch 2019). When these companies are hacked, it has a significant effect on the world’s economy and individuals’ privacy. Attacks are done through the Internet via the websites of online services, and they collect information such as passwords, credit card information, and security questions. Phishing attack (Gupta et al. 2017), which utilizes fake websites, emails, and free offers, is an example of a social engineering attack.

#### SCADA system and communication technologies in WAN

The SCADA system connects the control center, load substations, generating stations, and other service providers. It is responsible for both supervisory control and data collecting, as suggested by its name. Malware transmitted to the system via infected removable storage media and email attachments are a common threat. A Computer with WiFi capability and a WAN connection might potentially act as a link to the SCADA system. In addition, Data integrity attacks (e.g., manipulating sensor or control signals) and a DoS attack that results in prolonged loss of control or sensing signals could have major impacts if they cause operators to make incorrect decisions on a SCADA system (Sridhar and Manimaran 2010; Gao et al. 2010). The author of Kalluri et al. (2016) presents an examination of the impact of DoS on SCADA systems. Collecting the measurement data and transmitting the control signal at the correct moment in a SCADA system is extremely important and necessary for regulating, which is why RA and TDA represent big threats to the power system (Li et al. 2020). SCADA protocols and technologies used in WAN like Distributed Network Protocol-3 (DNP3), Modbus, and IEC-61850 are also susceptible to many attacks of wireless technologies such as MiTM attacks (Wlazlo et al. 2021).

#### The advanced metering infrastructure (AMI)

The AMI is a vital component in the deployment of the smart grid, facilitating bidirectional communication between electric utility providers and their consumers. The integration encompasses a range of components, such as the communication network, smart meters, and the MDMS. However, the AMI is susceptible to cyber threats and weaknesses, as emphasized in many scientific reviews (Kumar et al. 2019; Wei et al. 2018).

While conventional meters were vulnerable to physical assaults, smart meters provided novel avenues for CAs. Smart meters may possess restricted internal hardware and firmware due to their bulk procurement and cost-oriented design, frequently placing emphasis on cost rather than security factors (Wei et al. 2018). The situation presents a favorable circumstance for potential attackers. A possible type of attack is known as smart meter cloning, in which an adversary is able to replicate the identification of a meter or radio channel. This unauthorized replication grants the attacker the ability to manipulate power billing or falsely declare zero use (Kumar et al. 2019).

Moreover, the emergence of renewable energy technologies, such as solar and wind power, enables consumers to play the role of energy producers, hence facilitating the opportunity to sell excess energy back to utility providers. Nevertheless, it is possible for malicious individuals to take advantage of weaknesses within the system in order to influence the billing process for green energy units (McLaughlin et al. 2010). Other vulnerabilities have been identified in specific systems like the “442SR wind turbine,” where injecting malicious scripts can enable remote control of the turbine (CISA 2017).

In the context of a DR program, a demand response automation server sends load status information to consumers. However, in the event of hacked forwarding points, there is a possibility for the redirection of this sensitive information to unauthorized nodes, so affecting the privacy of customers (Paverd et al. 2014).

In addition, the AMI communication network, which establishes a connection between the HAN using protocols such as WiFi, Zigbee, or Z-wave, and then connects to the utility in WAN, is vulnerable to unwanted interception, eavesdropping, malicious code injection, and replay attacks (Kaplantzis and Şekercioğlu 2012; Saxena and Grijalva 2017; Vaidya et al. 2013). The presence of a large number of smart meter data collector devices inside the network creates a significant scale that may be exploited by attackers, potentially resulting in vulnerabilities. Moreover, the multi-hop communication system employed in the AMI is susceptible to DoS, which can occur when hacked nodes within the network deceive traffic by impersonating the shortest path, eventually leading to the loss of vital information (Kumar et al. 2019).

#### The transmission and distribution domains

The regular maintenance of high-voltage substations necessitates the implementation of electronic remote access. This is done to facilitate continuing data analyses, which are collected from the RTUs, IEDs, and PMUs. Simultaneously, it facilitates remote access by unauthorized individuals to the networks, especially in an unmanned IP-based substation (Bulbul et al. 2015). Although border technologies have the capability to limit remote access from certain IP addresses, they do not engage in extensive examination of the control and data content transmitted between the boundaries of two networks (Yamashita et al. 2020a). For instance, the implementation of IP-based IEDs has the potential to provide a security risk, since they might be susceptible to manipulation by malicious actors (Hong et al. 2014). The report (Yamashita et al. 2020b) highlights that the act of opening circuit breakers at substations through a switching attack can potentially be executed by compromising direct connections to IEDs.

Furthermore, other various data accumulating devices, such as RTUs and PLCs, can be utilized to enhance SCADA systems. RTU is a microprocessor-controlled electrical device that functions as a link between the SCADA and the outside world. A RTU is able to support multiple standard protocols (Cabus et al. 2022). Using multiple communication protocols introduces several potential vulnerabilities that can be exploited by an adversary to obtain sensitive information or even gain access to the system. Reference (Good 2020) describes several network potential attacks on the RTU Protocols.

Lastly, the PMU stands out as a critically significant device that is extensively employed in the SG. The primary objective of a PMU is to measure electrical values, including voltage, current, frequency, and phase angle, at different positions within the grid. These measurements play a crucial role in monitoring the system over time and identifying any anomalies that may occur. To facilitate this, the measurements are transmitted through the PMU communication network to a PDC. The communication between PMUs and PDCs takes place via phasor data concentrators on NASPInet-based wireless networks that utilize IP multicast routing protocols. However, the security of these networks becomes a concern when they are compromised by intruders. In such scenarios, the private domain networks responsible for transmitting synchrophasor measurements become vulnerable to FDIA (Wang et al. 2017). This compromise can have far-reaching consequences as each compromised network can be propagated on a larger scale through the use of malware agents. These agents automate the intrusion process and actively search for relevant synchrophasor information. Moreover, it is important to highlight that the IEEE C37.118 and IEC 61850-90-5 are widely recognized as two of the most popular PMU communication frameworks. The vulnerabilities associated with the IEEE C37.118.2 communication protocol can lead to various types of CAs, including Distributed Denial-of-Service (DDoS) attacks (Farooq et al. 2018). Additionally, PMUs are highly vulnerable to TSAs, where an attacker compromises the time reference of a group of PMUs, enabling them to manipulate the phase angle of the recorded phasors. This manipulation can have severe consequences on the operation and control of the grid (Zhang et al. 2013; Shereen et al. 2022). Furthermore, the propagation of tampered substations within a WAN has the potential to impact all synchronized substations, resulting in inaccurate metering, disruptions to state estimation, and significant socio-economic or operational impacts (Wang et al. 2017).

#### Automated generation control (AGC)

AGC depends on the SCADA telemetry system to provide tie-line and frequency measurements. A CA on AGC could result in significant consequences for system frequency, stability, and economic operation. The primary controller of AGC is vulnerable to the Aurora attack (Zeller 2011), wherein an attacker could quickly open and close the circuit breakers of a generator, causing it to become desynchronized and eventually damaged if the timing of such actions falls within a critical time window.

The main resources targeted by the DoS attack are the communication channels (linking RTU/PMU and the control center, and connecting the control center and governor). DoS attacks can delay the transfer of measurement data to the control center, impact the updating of the control command from the control center, and delay the control signals given to the actuator, so degrading the performance of the power system (Li et al. 2019). Another attack capable of corrupting the LFC system’s functionality, the main component of AGC can be launched by distorting data (e.g., FDIA) (Abbaspour et al. 2019) or by injecting delays (e.g. TDA Sargolzaei et al. 2013, 2014) into the telemetered measurement states or control signals. Further, the local control loops of the AVR and governor control are independent of the SCADA telemetry infrastructure as they rely on local sensing of the terminal voltage and rotor speed. However, these control loops are still susceptible to malware that may infiltrate the substation via other entry points, like USB keys.

## Solutions and countermeasures for cybersecurity in SG categorized based on NIST framework

**Fig. 5:**
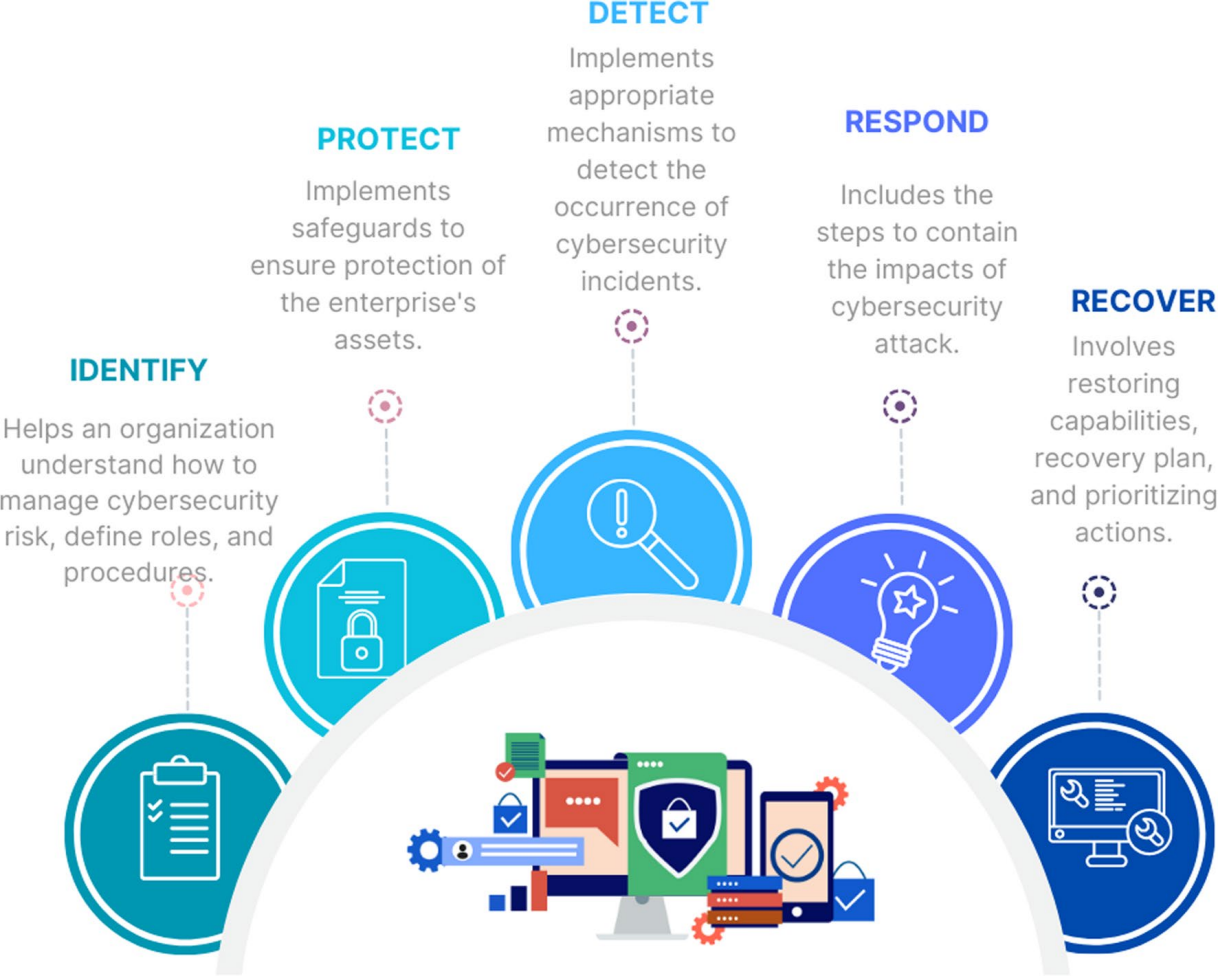
The five functions of the NIST cybersecurity framework

Today, cybersecurity threats have become more frequent and sophisticated, necessitating the implementation of effective risk management strategies by organizations. Utilizing a standardized framework for managing cybersecurity risks is a crucial method for achieving this objective. The NIST Cybersecurity Framework is a well-recognized and valuable tool that organizations from a variety of industries have adopted to enhance their cybersecurity risk management (Standards 2021). It provides a comprehensive set of best practices and guidelines for successfully addressing cybersecurity risks. The three primary components of this framework are Core, Implementation Tiers, and Profiles. As shown in Fig. 5, the Core consists of five concurrent and continuous functions (Identify, Protect, Detect, Respond, and Recover). It provides a strategic perspective on the cybersecurity risk management approach of an organization. By utilizing the NIST Cybersecurity Framework, organizations are able to identify and understand their cybersecurity risks, secure their assets and data, detect attacks, respond to and recover from incidents, and continually enhance their cybersecurity posture.

Numerous research efforts have been devoted to addressing cybersecurity in the SG. This section aims to provide a comprehensive overview of solutions and countermeasures proposed in different studies that can be implemented to enhance SG’s cybersecurity. To this end, we classify these solutions according to the five functions. Specifically, we investigate the use of BC-based techniques as a means of protecting the SG in the Protect function, while in the Detection function, we focus on the use of AI mechanisms. The most important categories of solution techniques covered in our study are listed in table 4. Additionally, it presents some of the mentioned solutions based on their potential application locations within the SG.

**Table 4:** Methods and countermeasures to defend against CA in SG classified based on NIST framework

Refrences	Targets	Attack Types	Techninques	Datasets, simulation tools	NIST function
Hahn and Govindarasu (2011)	AMI (SM,MDMS, ..), communication networks (Zigbee, WiMax)	SM tampering, authentication fraud	Graph theory	Southern California Edison’s (SCE), AMI Use Cases	Identify
Klaer et al. (2020)	Communication networks	Not specified	Graph theory	SGAM Toolbox	Identify
Nagaraju et al. (2017)	Not specified	Not specified	Fault trees (FT) and attack trees (AT)	Not specified	Identify
Chen et al. (2011)	SMs	Not specified	Petri net	Python	Identify
Girdhar et al. (2021)	EV charging stations	STRIDE model (tampering, spoofing, repudiation, information disclosure, DoS, and elevation of privilege)	Hidden Markov Model (HMM)	Not specified	Identify
Kim et al. (2011); Leszczyna (2019)	Communication networks	Not specified	Secure protocols and standards	Not specified	Protect
Rosic et al. (2013)	Not specified	Authentication fraud	Access control strategies	Not specified	Protect
Zavala-Díaz et al. (2021)	Not specified	MiTM and eavesdropping	Cryptography	Embedded devices (Raspberry Pi)	Protect
Laftimi et al. (2022)	MDMS server	Authentication fraud	Artificial intelligence, Blockchain	Solidity language with Ethereum Virtual Machine (EVM).	Protect
Wang et al. (2019)	The authentication process in edge-computing-based smart grid systems	Authentication fraud	Blockchain Technique	Hyperledger Composer, Docker Engine, Solidity and serpent languages	Protect
Badra and Borghol (2021)	The service provider (users’ information)	MiTM	Blockchain Technique	Not specified	Protect
Liang et al. (2018)	Communication networks, SMs, Sensors...	MiTM: data manipulation and eavesdropping (DME)	Blockchain Technique	The IEEE-118 system	Protect
Mengelkamp et al. (2018)	Energy market	Not specified	Blockchain Technique	Photovoltaic (PV) systems in Germany in 2013	Protect
Pop et al. (2018)	DR programs	MiTM (DME)	Blockchain Technique	The energy profiles of different United Kingdom (UK) buildings published by governmental agencies^1,2^, Solidity language.	Protect
Guan et al. (2018)	AMI (user’s electricity consumption)	Mitm (DME), authentication fraud	Blockchain Technique	Not specified	Protect
Jokar et al. (2013)	IEEE 802.15.4 networks	Spoofing attack	Localization techniques	Real office environment in the Lab of Electrical and Computer Department at the University of British Columbia.	Detect
Wang and Wyglinski (2016)	IEEE 802.11 networks	MiTM	Localization techniques	Backtrack5 tools, MATLAB	Detect
Delcourt and Le Boudec (2020)	Sensors	TSA and RA	Localization techniques	SIMULINK in MATLAB	Detect
Huang et al. (2018)	AGC	RA	Watermarking techniques	The Northeastern Power Coordinating Council (NPCC) 140-bus power system	
Li et al. (2017)	AMI	Data integrity attack	Statistical techniques	AMPds dataset	Detect
Kallitsis et al. (2016)	AMI	Several types	Statistical techniques	Not specified	Detect
Nezhad et al. (2016)	Communication networks	DoS,DDoS	Statistical techniques	R software environment, Box-Cox and ARIMA toolboxes, Darknet, CAIDA, and DARPA1998 data set.	Detect
Manandhar et al. (2014)	Sensors, communication networks, EMS	DoS, FDIA	Statistical techniques	IEEE 9-bus system, MATPOWER package in MATLAB	Detect
Wang et al. (2017)	Sensors	TSA	Artificial intelligence	IEEE 14-bus system, with real data from New York Independent System Operator (NYISO) ^3^	Detect
Al-Abassi et al. (2020)	Modbus packet	FDIA	Artificial intelligence	Datasets provided by Gas Pipeline and Secure Water Treatment ^4^	Detect
Sakhnini et al. (2019)	EMS	FDIA	Artificial intelligence	IEEE 14-bus, IEEE 57-bus, and IEEE 118-bus systems, MATPOWER	Detect
Zhang et al. (2020)	EMS	FDIA	Artificial intelligence	IEEE 13-bus system, MATLAB, Python	Detect
Xiong et al. (2022)	EMS	FDIA	Artificial intelligence	IEEE-14 bus, IEEE-39 systems, MATPOWER in MATLAB, Python	Detect
Yan et al. (2016)	EMS	FDIA	Artificial intelligence	IEEE 30-bus system, MATPOWER	Detect
Wang et al. (2021)	EMS	FDIA	Artificial intelligence	MATPOWER	Detect
Yang et al. (2021)	EMS	FDIA	Artificial intelligence	IEEE 14, 118-bus systems, MATPOWER	Detect
Yin et al. (2021)	EMS	FDIA	Artificial intelligence	SimBench dataset, Kubernetes and Docker software	Detect
Elsaeidy et al. (2020)	Communication networks	RA	Artificial intelligence	Dataset provided by collaboration between the University of Canberra and the Queanbeyan-Palerang Regional Council under the Commonwealth Government’s smart cities and Suburbs Program in 2017	Detect
Sriranjani et al. (2023)	ZigBee network	RA	Artificial intelligence	voltage and current sensors, Arduino, Zigbee and Raspberry Pi, Thing Speak, MATLAB	Detect
Zhe et al. (2020)	Communication networks	DoS	Artificial intelligence	KDD99 dataset	Detect
Gubbi et al. (2022)	All the components	malwares	Artificial intelligence	Over 100 programs, both good and bad	Detect
Zhang et al. (2022)	LFC	FDIA	Kalman filtering, AI	SIMULINK in MATLAB	Detect
Bi et al. (2021)	LFC	FDIA	Artificial intelligence	IEEE 39-bus system	Detect
Bi et al. (2021)	LFC	DoS	Artificial intelligence	SIMULINK in MATLAB, Box and Jenkins gas furnace dataset and Mackey-Glass dataset	Detect
Demir et al. (2018)	Not specified	DDoS	Isolation affected systems techniques, Cloud-based techniques	Amazon’s EC2, PlanetLab test-bed	Respond and Recovery
Rahiminejad et al. (2023)	AGC	Not specified	Cyber-Physical Multi-Aspect Resilience-Based Recovery Metric (CPARM)-based	The 39-bus New England test system, IEEE 30-bus system	Recovery

^1^https://www.data.gov.uk/dataset/da9a88d6-6535-4c7f-8d54-a93a50b2f177/the-national-archives-energy-consumption

^2^https://www.data.gov.uk/dataset/fee711fd-b405-4939-8945-5f9189839ad0/department-for-education-gas-and-electricity-half-hourly-data

^3^https://www.nyiso.com/energy-market-operational-data

^4^https://hdl.handle.net/11668/20006

### The identify function

The Identify function is the initial stage in the five-step framework that concentrates on comprehending an organization’s cybersecurity risk situation. This function involves gaining a clear understanding of the business context of the organization, the critical systems, and devices, as well as identifying the potential cybersecurity risks and vulnerabilities associated with them. As part of a risk assessment, vulnerabilities can be identified and documented. The Asset Management subcategory within this function is responsible for identifying and managing the devices, systems, data, personnel, and facilities that support the organization in achieving its business objectives. This is done by prioritizing the assets based on their relative significance and the organization’s risk strategy. Whereas Risk Assessment is responsible for evaluating identified risks. The methodology is iterative and dynamic, allowing it to be modified as new threats emerge, new vulnerabilities are identified, and the impact of cyber assaults on the SG changes. This phase involves analyzing the physical, economic, and social effects of a successful CA.

In the article (Faheem et al. 2018), the authors emphasized the essential quantitative and qualitative requirements that the infrastructure must meet in various applications within the smart grid. The study of Quality of Service (QoS) requirements and applications in the SG, including factors such as latency, bandwidth, data rates, throughput, and reliability, significantly contributes to enhancing cybersecurity, particularly in identifying potential cybersecurity risks within the SG environment. By considering these QoS factors, organizations can assess the performance and resilience of the SG infrastructure, identifying vulnerabilities that may be exploited by cyber attackers. Understanding and meeting latency requirements allows for timely response to security events, while sufficient bandwidth and high data rates ensure efficient and secure data transmission. Additionally, reliable systems with adequate throughput reduce the potential for disruptions and failures that can be targeted by cyber threats. By studying and addressing these QoS requirements, organizations can implement appropriate security measures, risk mitigation strategies, and proactive monitoring, ultimately bolstering the overall cybersecurity posture of the smart grid. For instance, the wireless networking solution in the SG empowers control and management competencies, resulting in benefits such as cost reduction, enhanced electricity quality, increased production speed, improved flexibility, and simplified installation (Mahmood et al. 2018). However, wireless channels in the smart grid face unique challenges, including fading, multi-path effects, equipment noise, heat, electromagnetic interference, and dusty environments. Consequently, the reliability of wireless links between sensors in SG applications can vary across different locations and time periods, making it challenging to achieve QoS aware multi-hop data transmissions for WSN-based SG applications (Faheem and Gungor 2018). To address these challenges, the authors in Faheem et al. (2019) propose a novel channel-aware distributed routing protocol called CARP for SG applications. CARP incorporates a cooperative channel assignment mechanism that significantly improves detection reliability and mitigates noise and congestion in spectrum bands, resulting in reliable and high-capacity links for SG applications. Additionally, CARP’s multi-hop routing mechanism selects secondary user relay nodes with abundant spectrum information and a longer ideal probability of low interference to support higher capacity data requirements and maximize spectrum utilization.

The article (Hahn and Govindarasu 2011) proposes a framework that considers the physical impacts of CAs on the SG. The framework consists of a risk assessment methodology, a modeling approach to represent SG components and their interconnections, and a simulation tool to demonstrate the consequences of CAs on the SG. The objective of attack modeling is to identify ways by which attackers could exploit vulnerabilities. Klaer et al. (2020) presents a graph-based modeling approach to depict the electronic and physical components of SG architecture. The model can be used to identify the system’s critical components and assess the potential impact of the attacks. For the same goal, Nagaraju et al. (2017) gives an overview of fault and attack tree modeling and their applications in cybersecurity risk management. Fault trees are a graphical representation of the logical connections between events and conditions that can contribute to system failure. The authors define attack trees as a hierarchical representation of the actions the attacker must take to accomplish a particular objective. In addition, they discuss the varieties of attack trees, such as sequential and parallel attack trees. In sequential attack trees, a hacker must complete a series of steps in a specific order to compromise the system, whereas, in parallel attack trees, multiple steps can be performed simultaneously. Similarly, Petri nets are a form of graph-based modeling approach that are used to simulate the behavior of complex systems, such as the cyber and physical interactions of the SG (Chen et al. 2011). It is also necessary to mention in this area, the STRIDE-based threat modeling, which is a technique for identifying and analyzing potential threats to a system by analyzing six threat categories: tampering, spoofing, repudiation, information disclosure, DoS, and elevation of privilege. It is applied in Girdhar et al. (2021) in addition to Hidden Markov Model (HMM), a statistical model, to analyze and identify potential threats to the system of fast charging stations, and to model security attacks for a given range of identified attack vectors. Identified attack vectors are specific methods or techniques that attackers can use to exploit system vulnerabilities or deficiencies.

In addition to the mechanisms mentioned above in this field, there are further considerations to enhance protection in the SG. Multiple organizations have intensified their efforts to enhance cybersecurity by creating frameworks and guidelines with specific recommendations for various aspects of the SG. One notable example is the Electric Reliability Organization (ERO) Enterprise, which comprises the NERC and six regional reliability entities. The ERO Enterprise has introduced the Cyber-Informed Transmission Planning Framework (CITPF), which serves as a roadmap for integrating cyber security into transmission planning activities within the smart grid (Corporation 2023). In this context, Transmission Planning is the process of modeling and studying the outage of elements in the bulk power system (BPS) and assessing the system’s performance under various contingencies. It involves identifying potential risks and vulnerabilities, analyzing system reliability, and recommending appropriate mitigations. The CITPF is a concept introduced by the ERO to integrate cyber security into the transmission planning process. It provides a roadmap for incorporating cyber security threats, particularly coordinated attacks, into transmission planning studies conducted by Transmission Planners (TPs) and Planning Coordinators (PCs). His goal is to improve the reliability and resilience of the BPS by considering cyber security risks in long-term planning assessments. By integrating cyber security into transmission planning, the framework aims to identify potential vulnerabilities and develop strategies to mitigate the risks associated with cyber attacks on the grid.

The CITPF consists of several steps. First, TPs define coordinated attack scenarios, focusing on aggregate risk resulting from common security control gaps. Next, they collaborate with design engineers and security professionals to translate attack scenarios into planning assessments by identifying potentially affected BPS elements. TPs then conduct planning studies, utilizing models, tools, and criteria to analyze BPS performance under the defined attack scenarios. The outcomes of these studies are analyzed by TPs, design engineers, and cybersecurity professionals to identify any reliability issues and develop a corrective action plan, which may involve additional cybersecurity controls or infrastructure improvements. Finally, necessary risk mitigations are implemented through collaboration between cyber security and design engineering teams, aiming to eliminate the credibility or feasibility of potential attack scenarios in future studies. The CITPF can be considered as part of the countermeasures against cyber attacks in the smart grid, specifically in the Identify function of the NIST framework. It helps in identifying critical assets, cybersecurity risks, and developing risk management strategies for transmission planning.

In general, these methodologies and tools can assist organizations in prioritizing risks, developing mitigation strategies, and ultimately enhancing the cybersecurity posture of SG.

### The protect function

The Protect function contributes to the prevention of potential cybersecurity incidents. It consists of the development and implementation of appropriate security controls to protect against identified cybersecurity threats and vulnerabilities.

In SG networks, secure protocols play crucial roles in ensuring the security and integrity of data transmission. The authors of Kim et al. (2011) proposed a scalable and secure transport protocol for SG data collection. Several studies also introduced and discussed SG standards as effective network CA countermeasures. For example, the authors of Leszczyna (2019) identified 19 standards that specify cybersecurity controls applicable to SG infrastructure. The advantages of using protocols and standards in SG security include interoperability, compliance, and improved security through guidelines and best practices. However, the adoption of new standards may be slow, and compatibility issues with legacy systems may require additional investment. Additionally, vulnerabilities of protocols can become public knowledge and can be exploited by attackers.

There are numerous effective strategies for administering SG networks and determining user access privileges. These strategies primarily manage permissions and provide enterprise assurance via a scalable solution. Numerous research studies have been conducted on access control measures in SGs. Rosic et al. (2013) proposes a role-based access control model that supports regional division in SG systems to improve security and efficiency, and the model is evaluated through simulation experiments.

Analyzing the requirements and quality of services, as well as developing specific mechanisms to meet those requirements, is crucial for enhancing the protection mechanism against cyber attacks in the SG. For example, in the context of real-time monitoring and control of the smart grid for continuous and quality-aware power supply in smart cities, an advanced QoS-aware communication framework is essential. The authors in Faheem et al. (2019) present a data-gathering scheme that utilizes the Internet of software-defined mobile sinks (SDMSs) and wireless sensor networks in the SG. Through extensive simulations, the designed scheme demonstrates superior performance compared to existing approaches. It successfully achieves its defined goals for event-driven applications in the smart grid. By developing and implementing robust systems, organizations can ensure reliable and secure communication, efficient data transfer, and effective response capabilities.

Cryptography and authentication are additional fundamental countermeasure techniques for SGs. Yu et al. (2022) proposes a lightweight identity-based secondary authentication scheme for the SG. Zavala-Díaz et al. (2021) presents an analysis of the cryptographic techniques implemented in embedded devices for SGs. Using cryptographic and authentication methods in SG systems can provide confidentiality, integrity, and authentication, helping to prevent unauthorized access and CAs. However, the use of cryptography can impact the performance of the system, leading to delays in data transmission and processing, and authentication can impact the user experience, requiring additional steps for users to access the system. In recent years, BC has been widely discussed and has demonstrated enormous potential in preventing various CAs on SGs, especially in cryptography and authentication. Following is a discussion of BC-based countermeasures.

- Blockchain-based cybersecurity techniques

BC is intended to facilitate peer-to-peer electronic payments directly, without the need for a trusted third party. BC is essentially a distributed, redundant, chain-connected, shared ledger database in which each network node is fault-tolerant and capable of point-to-point communications. The authors of Dong et al. (2018); Samy et al. (2021) take advantage of BC’s features and propose BC-based SG, and cyber-physical infrastructure models. BC can be used as an automatic and trusted authentication system for SG network services, preventing data tampering. The architecture proposed in Laftimi et al. (2022) aims to enhance the authentication process by incorporating BC technology and AI into the existing system. Wang et al. (2019) present a BC-based authentication and key agreement protocol for edge computing in SG.

In addition, BC can be considered a distributed database system, and two common data management applications are examined: stored data protection and data aggregation. The authors of Liang et al. (2018) designed a BC-based distributed information collection and storage mechanism. When a user registers for the Utility system on the registration page, they need to provide their name and share sensitive information like a key identifier with the service provider. The authors of Badra and Borghol (2021) suggested using BC to store this personal information. In Aggarwal et al. (2018), a BC model is proposed for securely preserving and accessing the data generated by customers. Guan et al. (2018) propose a privacy-preserving data aggregation scheme in which users are divided into distinct groups, and each group has a private BC to store the data of its members.

Furthermore, BC was used to manage SG operations, particularly the supervision of energy market services. The concept of consensus-based validation in the BC is introduced for the substantiation of DR programs (Pop et al. 2018). This improves the performance of the smart infrastructure. The work in Mengelkamp et al. (2018) implemented a local energy market utilizing a private Ethereum BC and a decentralized energy exchange open-source project. The researchers in Mylrea and Gourisetti (2017) utilized smart contracts to define the threshold values at which energy is bought or sold, as well as the exchange cost.

Using BC in SG security can provide a decentralized and distributed approach to security, ensure the integrity of data through an immutable ledger, and prevent tampering or manipulation. However, it may not be scalable enough to handle the large amounts of data generated by the SG system, and it can be energy-intensive. It generates redundant information, and each node needs to participate in every transaction’s verification process, leading to extra storage space consumption and high storage costs. Additionally, the processing time required to maintain the BC ledger can be particularly problematic in real-time data processing for maintaining system operations.

Game theory, a mathematical process that models strategic competition, has been widely adopted across various disciplines due to its effectiveness in analyzing security measures. It provides valuable insights into protecting smart grids from cyberattacks. By integrating game theory into security problems, the dynamics between attackers and defenders can be effectively addressed (Masum 2023). For instance, the authors in Wang et al. (2016) apply game theory in the modeling of attack-defense dynamics for power transmission grids. Defenders can minimize the expected loss of load and generator tripping by adjusting load generation based on current conditions. Attackers, conversely, aim to maximize their payoff by targeting specific points within the power operation network. The boundary between these two forces is presented in the dynamic game model. In the proposed model, defenders thoroughly consider the potential sequential actions carried out by attackers. They make sequential and repeated decisions while ensuring adherence to various constraints, such as power balance, ramping rates of generators, upper and lower limits of generators, and upper and lower limits of power flows on lines. Moreover, they take into account the altered system topology and the corresponding operating constraints that may arise following potential compromises. Another example pertains to the confidentiality of AMI where (Ismail et al. 2014) propose a game model that addresses two key aspects: how attackers choose their targets to gather maximum consumer data and how defenders determine the encryption level of outbound data on each device in the AMI. Furthermore, to address the CIA issues in the AMI, Abercrombie et al. (2014) present a Dynamic Agent-Based Game Theory (ABGT) approach. By selecting specific failure scenarios from the cyber security and impact analyses developed by NESCOR, they decompose the scenarios and model the interactions between attackers and defenders as a two-player stochastic game, and then Nash Equilibriums, are computed to determine the optimal defense strategies.

Furthermore, in relation to the mechanisms discussed in this step, there are additional factors to be taken into account in order to enhance protection in the smart grid. Various organizations worked closely together to develop and offer recommendations and tools to enhance the protection phase in the smart grid. In line with this, the ERO in the CITPF (Corporation 2023) includes a list of necessary risk mitigations to prevent coordinated attacks. These recommendations encompass enhancements in infrastructure, controls and protections, operating procedures, and the cyber security program. For instance, in the case of an unauthorized remote access attack, the framework suggests several mitigating cyber security controls. These include on-demand session authorization, malicious code detection, authentication, session logging, monitoring, termination, and change control/baseline monitoring. Furthermore, the Cybersecurity and Infrastructure Security Agency (CISA) provides additional resources to reduce cyber attack surfaces and vulnerabilities, thereby enhancing the overall cyber security posture of organizations. These resources include the freely available CSET tool for evaluating security posture, the KEV Catalog to track actively exploited vulnerabilities, and Cyber Hygiene Vulnerability Scanning for internet-facing services. Moreover, CISA offers a Validated Architecture Design Review based on NIST standards and industry best practices. This assessment can be conducted on both information and operational technology infrastructures in the SCADA systems. Additionally, the S.O.S guide, which stands for “Get your Stuff Off Search,” provides guidance on reducing the attack surface of Internet-facing devices (Corporation 2023).

### The detect function

The Detect Function facilitates the detection of cybersecurity incidents in a shorter time. Real-time awareness and continuous system monitoring are essential for detecting CA. In this section, potential countermeasures against attacks on SGs are outlined, particularly in terms of AI, which has been extensively implemented in SGs due to its strong capacity to extract useful information.

First, Jokar et al. (2013) presents a method for detecting deception in static IEEE 802.15.4 networks based on the spatial correlation property of the received signal strength (RSS). Similarly, the authors of Wang and Wyglinski (2016) propose a received signal strength indicator (RSSI)-based detection mechanism for MiTM attacks. The authors of Delcourt and Le Boudec (2020) propose a Time Difference of Arrival TDOA-localization technique that is resistant to TSA. These localization-based approaches to detect attacks in SGs can quickly identify the location of an anomaly, allowing operators to isolate and mitigate its effects. However, these techniques have limited network coverage and may not be able to detect attacks outside of the monitored areas. Then, Numerous CA detection algorithms are based on the technique of watermarking in order to detect malicious actions during RA (Romagnoli et al. 2019). It involves embedding a unique signature within the data to verify the authenticity and detect any tampering that may have occurred. In Huang et al. (2018) an online watermarking algorithm is proposed to detect RA on AGC systems. The watermark is embedded in the control signal and extracted at the generator side to detect RA. Porter et al. (2020) proposes a dynamic watermarking technique that embeds the watermark signal in the system’s input signal. The watermark varies over time and is extracted at the system’s output to detect RA. These papers demonstrate the potential of watermarking techniques in detecting RA and MiTM in SGs. However, the effectiveness of these techniques may depend on the specific application and system being monitored. Watermarking can be computationally intensive and may increase system overhead, so it is important to carefully design and optimize the watermarking scheme to balance the trade-off between security and system performance. Moreover, Kallitsis et al. (2016) introduces an adaptive statistical approach to detect malicious intrusion attacks, that can compromise vulnerable meters and manipulate their readings. The method utilizes cumulative sum and exponentially weighted moving average algorithms, to detect sudden changes in sensor readings. Nezhad et al. (2016) proposes a method for detecting DDoS attacks. The method extracts features from the network traffic and builds a time series. An Autoregressive Integrated Moving Average (ARIMA) model is used to predict the number of packets, and the chaotic behavior of the prediction error time series is examined using the Maximum Lyapunov Exponent (MLE) to classify normal and attack traffics. ARIMA model employs statistical analysis and time-series data to analyze the information and forecast future values, while the MLE is a measure used to quantify the rate of divergence or convergence of nearby trajectories in the system (Franchi and Ricci 2014). Another approach based on prediction technique is proposed in Manandhar et al. (2014) by developing a detection algorithm that uses the Kalman filter (KF) to estimate the expected behavior of the system and analyze deviations using the chi-square test or Euclidean detector to detect faults and attacks. One advantage of these techniques is their ability to predict future behavior and state, which can be useful for systems, however, they may require a significant amount of data and computational resources to be effective, and poor data quality can lead to inaccurate forecasts and false alarms.

Despite the previously mentioned techniques, AI-based techniques have emerged as a prominent research area in this field due to their potential to provide real-time detection and adaptability to evolving threats. In this context, the next paragraph focuses on AI-based countermeasures.

- Artificial intelligence based cybersecurity techniques

The SG’s AI security-based techniques are becoming increasingly apparent. SG systems’ reliability and stability can be enhanced by employing AI techniques. Wang et al. (2017) create a machine learning (ML) classifier for TS attack detection. It suggests that artificial neural networks (ANNs) are a feasible option for implementing this detector. Methods of ML are also utilized in the malware detection method in Gubbi et al. (2022). The authors in Elsaeidy et al. (2020), Sriranjani et al. (2023) created models based on Convolutional Neural Network (CNN) and Support Vector Machine (SVM) for RA detection in SGs.

In the context of FDIA in a SG, numerous ML techniques have been implemented (Chen et al. 2017). Bitirgen and Filik (2023) proposes an approach for optimizing CNN, Long Short-Term Memory (CNN-LSTM) with Particle Swarm Optimization (PSO) to detect FDIA in the SG system. In Al-Abassi et al. (2020) Deep Neural Network (DNN) and Decision Tree (DT) detection models designed specifically for FDI and DoS detection are proposed. Ozay et al. (2015) has evaluated and compared more FDI attack detection algorithms. This study employs the supervised learning algorithms SVM and K Nearest Neighbor (KNN) and showed that KNN is more sensitive to system size and may perform better in smaller systems. Additionally, Yan et al. (2016) evaluated SVM, KNN, and Extended Nearest Neighbors (ENN) on the IEEE 30-bus system and compared their accuracy. All three detector designs have the capability to achieve optimal detection performance when faced with FDIA. In Zhang et al. (2022), the identical algorithms are combined with the KF algorithm. Sakhnini et al. (2019) proposes a detection method that combines supervised learning with three different feature selection (FS) methods in order to enhance the performance of the classification algorithm for FDIA in SG. The three algorithms used are SVM, KNN, and ANN. Binary Cuckoo Search (BCS), Binary Particle Swarm Optimization (BPSO), and Genetic Algorithm (GA) are the three FS techniques. The classification results indicate that SVM with GA was the most accurate among the three systems. Similarly, Xiong et al. (2022) presents an SVM detection algorithm that enables real-time FDIA detection in SG by employing the Uniform Manifold Approximation and Projection (UMAP) algorithm to accomplish effective feature extraction and dimension reduction of measurement data. The UMAP is a high-performance dimension reduction algorithm proposed in McInnes et al. (2018). Furthermore, Wang et al. (2021) demonstrates an efficient two-level FDIA detection scheme using the KF and Recurrent Neural Network (RNN). The first level consists of two base learners, the KF for state prediction to linear data and the RNN for the nonlinear data features. Using the fully connected layer and backpropagation module, the second-level learner then combines the results of two base learners. Moreover, Kurt et al. (2018) formulate the online attack anomaly detection problem as a partially observable Markov decision process (POMDP) problem and propose a model-free reinforcement learning (RL) algorithm for POMDPs. Using a multilayer perceptron classifier, Chen et al. (2017) also proposes a detection approach taking into account FDIA.

All of the above-mentioned FDIA detection mechanisms, concentrate on direct current state estimation. However, Yang et al. (2021) propose an alternating current FDIA detection method based on LSTM-Autoencoder. In addition, Ghazizadeh et al. (2023) presents a method for identifying LR attacks, which is a particular form of FDIA. The fundamental exploitable structure of the detection mechanism is based on analyzing estimated load data via the EMS and a deep LR.

On the other hand, Meriaux et al. (2022) compares how the detection of DDoS attacks, one of the most prevalent types of CA, on smart networks varies depending on: the ML method used for detection, the various datasets used for training, and the dataset features incorporated into the training. DT, Random Forest (RF), Quadratic Discriminant Analysis (QDA), SVM, Nave Bayes, and Extreme Gradient Boosting (XGBoost) are the various ML techniques utilized in this study. Similarly, in Zhe et al. (2020), the researchers test the SVM, DT, and Naive Bayesian Network classification algorithms on the KDD99 dataset, and the SVM model appears to be the most effective. Additionally, the article (Li et al. 2019) proposed a data prediction-based method as a defense against the DoS attack on LFC. Combining the deep learning (DL) algorithm and the Extreme Learning Machine (ELM) algorithm, the proposed method benefits from the fast speed of the ELM and the high accuracy of the DL. Based on this, the authors are able to detect and replace lost data, assure the normal operation of the LFC system, and thus prevent DoS attacks.

In conclusion, the use of AI-based techniques in attack detection can provide several advantages in SG security. AI algorithms can analyze large amounts of data from various sources in real-time, detecting and responding to attacks quickly and accurately. However, these techniques may have limitations depending on data availability. It is worth mentioning that these methods require an appropriate dataset to test and implement their algorithms; then, for security reasons, it is not always possible to work with real data in SGs. In addition, implementing AI-based detection systems can be costly, especially for smaller utilities with limited budgets.

### The respond function

The Respond Function aids in mitigating the effects of a potential cybersecurity incident. Communication and response must be quick and efficient. In the SG context, this involves implementing several stages for responding to CAs. First of all, response planning is essential to ensure that appropriate actions are taken promptly. This includes creating and maintaining a response plan document that identifies key stakeholders and defines their roles and responsibilities.

Then, it is necessary to establish effective communication protocols for notifying relevant stakeholders, including law enforcement and external parties as necessary. So, to keep all parties informed, communication should be maintained during and after an incident.

Once an attack detection is in place, the next step is to establish an analysis to identify all affected assets and data and their impact on the organization. This information is crucial in ensuring an effective response and triggering appropriate recovery activities.

Furthermore, to prevent the incident from spreading and causing further damage, mitigation measures should be implemented. This may involve isolating affected systems, disabling affected services, or applying patches to vulnerable systems. For instance, the article (Demir et al. 2018) proposes a mechanism to quickly quarantine malicious clients during DDoS attacks in a cloud-assisted SG system. The mechanism isolates the suspected malicious clients by redirecting their traffic to a quarantine server while allowing legitimate traffic to continue to flow. It uses a local cache and pre-fetching technique to minimize the delay in redirecting traffic, resulting in a notably short containment time.

Lastly, improvements should be made by incorporating lessons learned from current and previous detection/response activities to improve the overall resilience of the SG system. Conducting a post-incident analysis is an example of this step, which can help identify areas for improvement in the response plan and implement changes to enhance the organization’s ability to respond to future incidents.

### The recover function

The Recover Function’s activities facilitate the fast return to normal operations. it plans and implements the relevant activities to maintain resilience and restore any affected capabilities or services. Several studies have explored the context of SG recovery.

For example, a strong proactive DDoS attack defense mechanism and recovery strategy is proposed in Demir et al. (2018), which dynamically changes the open ports of the broker servers to efficiently drop the invalid packets in the firewall. Furthermore, it diffuses consecutive data packets over several servers versus a single server to rapidly recover the attacked system in the cloud.

In a different approach, Rahiminejad et al. (2023) proposes a Cyber-Physical Multi-Aspect Resilience-Based Recovery Metric (CPARM)-based CA recovery strategy. The suggested technique examines attack effects before physical consequences and smart multi-stage attacks. Four operational characteristics related to physical-side resilience: load demand, reserve capacity, line capacity, and power system reliability are examined. Cyber-side resilience is also included depending on the maximal physical impact the attack may have, whether the attackers succeeded or not. The suggested recovery approach takes into account the SG capabilities and limits, especially the AGC.

Furthermore, after taking substation control, malevolent attackers can trip all transmission cables to stop power delivery, leaving transmission line-connected regions asynchronous. Transmission lines are closed automatically or manually to restore electrical flow after the attack, but equipment can be damaged. Reclosing time must be carefully adjusted to reduce these impacts. Wei et al. (2019) offers a recovery method to reclose tripped transmission lines at the right time. A deep RL framework makes real-time decisions and adapts to unpredictable CA scenarios.

On the other hand, because rapid restoration of the power supply following an interruption could significantly reduce the outage loss, Liao et al. (2019) suggests an improved two-stage optimization method for network and load recovery during power system restoration, consisting of a mixed-integer linear programming model and a continuous non-linear optimization method based on AC power flow.

## Open issues and research challenges

While there have been significant research efforts in the field of SG cyber-physical security, there are still several challenges that require further attention. This section highlights critical challenges and opportunities for future research in this area.

- Firstly, additional research is required in attack prevention and detection methods to develop advanced communication technologies that can transfer data securely in a specific part of the SG without affecting the reliability (Sridhar et al. 2011). Ding et al. (2022) indicates that additional effort is required to develop higher-level algorithms to detect attacks specifically targeting AGC and AMI. The review (Kawoosa and Prashar 2021) highlights the need to provide dynamic and customized cybersecurity solutions for AMI. This is due to the fact that SMs require real-time communication and have limited computational resources.
- Secondly, the dependability of components used in data sensing and communications needs to be investigated to analyze the impacts of each attack and enhance the method of attack isolation. For future research in this area, interdependence needs to be comprehensively explored, and the modeling approaches can be applied. Additionally, in the article (Zhang et al. 2021), the authors emphasize the importance of comprehensively studying the interdependence between the cyber and physical layers in the SG. This entails developing realistic cyber-physical simulation software and exploring the interdependence with other critical infrastructures such as communication, water, and transportation networks.
- Thirdly, while several studies have explored the use of AI methods to detect attacks in power systems, incorporating more external accurate factors for feature enhancing or combining AI with other techniques can lead to more efficient decision-making. Additionally, we also need to take into consideration the issue of high implementation costs for these types of solutions, as well as the absence of real datasets which can lead to inaccurate results in research.
- Fourthly, more research is needed to address the complex cybersecurity challenges facing the SG system in the context of cloud and quantum computing. Cloud computing is becoming increasingly important in the SG, but it also increases the risk of CAs. Then, developing new cryptographic algorithms that can withstand quantum attacks or exploring the use of quantum computing to improve the security of SG systems are new research questions. The article (Bera et al. 2014) focuses on cloud applications in the SG and identifies future opportunities for cloud-based energy management. However, the article also highlights research challenges, such as exchanging energy information between the cloud and the SG. The authors note that allowing grids to exchange energy with cloud energy storage devices, especially in the presence of intrusion, is an important issue that needs to be addressed.
- Then, at the end of their study, the authors (Kayastha et al. 2014) presented a significant challenge, how to ensure secure data sensing and communications in the SG while optimizing the cost of network design. Public networks such as cellular networks and the Internet may make the SG vulnerable to cyber threats, so private networks are a more secure option, but not always economical. A hybrid approach, where noncritical applications use public networks and critical applications use private networks, may be considered. Additionally, before deploying network infrastructure, a cost-benefit analysis should be performed to balance the cost of equipment installation and maintenance with performance metrics such as latency, loss, and bandwidth. For example, using CR techniques to save on wireless bandwidth may result in packet loss and delay, so the tradeoff between using CR and dedicated wireless channels must be investigated to minimize total cost.
- Furthermore, IoT devices hold great promise for digital transformation, including in the power grid infrastructure. However, in SG infrastructure, IoT security remains challenging (Borgaonkar et al. 2021) due in part to tradeoffs between cost and performance, which can increase the attack surface for potential cyber threats. Other challenges include the limited processing power and storage of many IoT devices, the lack of standardized security protocols, and the need to protect users’ data privacy, as IoT devices collect large amounts of data about their behavior.
- Additionally, while several organizations are working to develop standards for the SG system to improve interoperability, further research is needed in this area. Proposing mechanisms to link devices with different protocols, such as using middleware or application programming interfaces, can also address interoperability problems.
- Finally, the NIST emphasizes the equal importance of all five functions within their framework and recommends achieving a balance among them. However, prior academic works have not adequately addressed this balance. Our study addresses this gap by highlighting the existing deficiencies in scientific research regarding the different functions of the framework. Specifically, we identify a clear need for more extensive research in the areas of response and recovery mechanisms, which are crucial components for achieving an effective cybersecurity approach.

In conclusion, identifying and tackling the critical challenges outlined in this section can serve as a starting point for research in this field. Additionally, addressing these gaps can improve the cybersecurity, reliability, and interoperability of the SG system.

## Conclusions

This paper provides a comprehensive overview of the SG architecture, communication networks, and the different CAs that can target the system. The most important equipment of the smart grid is visualized in a diagram that includes descriptions of the links and dependencies between them. This provides a better understanding of the dependability of components and the effects of attacks on the system. The paper presents different communication technologies in the context of SG networks, including HANs, NANs, and WANs. It also reviews an analysis of attacks, categorizing them based on their type and target points. Additionally, it includes a countermeasures list classified based on the NIST Cybersecurity Framework. Our study sheds light on the importance of achieving a balance among the five functions. While prior academic works have overlooked this balance, our research has identified the deficiencies in addressing the different functions of the framework. As a final point, it emphasizes critical challenges and opportunities for future research, such as the need for extensive research in response and recovery mechanisms and for customized advanced communication technologies and attack detection techniques for specific parts of the SG like AMI. Addressing these challenges can create new research opportunities to improve the cybersecurity, reliability, and interoperability of the SG system.

## Data Availability

Not applicable.
